# African swine fever virus pMGF110-9L plays a critical role in the integrated stress response and viral pathogenesis

**DOI:** 10.1128/jvi.01080-26

**Published:** 2026-09-08

**Authors:** Shuai Fan, Han Zhong, Dan Li, Jing Zhang, Yuhang Zhang, Man Hu, Wenrui He, Bo Wan, Zixiang Zhu, Shichong Han, Haixue Zheng, Gaiping Zhang

**Affiliations:** 1 International Joint Research Center of National Animal Immunology, College of Veterinary Medicine, Henan Agricultural University 731518, Zhengzhou, China; 2 State Key Laboratory for Animal Disease Control and Prevention, Lanzhou Veterinary Research Institute, Chinese Academy of Agricultural Sciences 111658, Lanzhou, China; 3 Longhu Laboratory, Henan Agricultural University, Zhengzhou University 70573, Zhengzhou, China; 4 Ministry of Education Key Laboratory for Animal Pathogens and Biosafety, Henan Agricultural University 70573, Zhengzhou, China; Northwestern University Feinberg School of Medicine, Chicago, Illinois, USA

**Keywords:** African swine fever virus, pMGF110-9L, integrated stress response, eIF2α phosphorylation, ER stress, translational shutdown, stress granules

## Abstract

African swine fever virus (ASFV) is the causative agent of ASF, a highly contagious and frequently fatal hemorrhagic disease in domestic swine and wild boars, posing a significant threat to the global pig industry. To ensure successful replication, ASFV may modulate cellular stress mechanisms to hijack the host translation machinery and promote viral factory formation to favor viral protein synthesis and counteract host defenses. However, the interplay between ASFV replication and cellular stress responses has not yet been elucidated, hindering the development of vaccines or therapeutic options. In this study, we dissected how ASFV manipulates the integrated stress response (ISR) to facilitate viral replication and identified the virulence-related factor pMGF110-9L as the strongest activator of ATF4 expression, a key factor downstream of eIF2α phosphorylation during ISR activation. We characterized pMGF110-9L as an endoplasmic reticulum (ER)-localized membrane protein, the expression of which profoundly reorganized the cellular compartments, leading to ER swelling and aggregation. Further proteomic analyses and biochemical experiments revealed that pMGF110-9L is crucial for ASFV-induced ER stress and promotes the activation of the PERK-eIF2α-ATF4, IRE1α-XBP1, and ATF6 branches of the unfolded protein response. Specifically, we demonstrated that pMGF110-9L activates the ISR through the PERK/PKR-eIF2α pathway, leading to viral takeover of the protein synthesis machinery, accompanied by global protein synthesis shutdown and stress granule formation. Furthermore, the transmembrane domain of pMGF110-9L is indispensable for targeting the ER and subsequent stress responses. Overall, these findings inform novel insights into ASFV–host interactions and define a previously uncharacterized role for pMGF110-9L in ASFV pathogenesis.

**IMPORTANCE:**

African swine fever (ASF), caused by African swine fever virus (ASFV), poses a catastrophic threat to global pork production. The complex pathogenesis and immunoevasion strategies of ASFV have not been clearly elucidated, raising safety concerns regarding modified live virus (MLV) vaccines. Therefore, a better understanding of how ASFV manipulates host responses to establish intracellular conditions favorable for viral replication could provide essential insights into ASFV–host interactions and enable the development of safer and more effective MLV vaccines. In this study, we comprehensively elucidated the relationship between ASFV-encoded proteins and the activation of the integrated stress response. We identified pMGF110-9L as a prominent virulence factor involved in viral takeover of the protein synthesis machinery, accompanied by endoplasmic reticulum (ER) stress, ER-associated aggresome formation, halted host protein translation, and stress granule formation. Importantly, the transmembrane domain of pMGF110-9L was critical for triggering cellular stress mechanisms. This study advances our understanding of ASFV pathogenesis and highlights pMGF110-9L as a potential target for MLV vaccine development.

## INTRODUCTION

African swine fever (ASF) is a highly contagious and frequently fatal hemorrhagic disease caused by the ASF virus (ASFV), primarily affecting domestic pigs and wild boars. The disease manifests with fatality rates approaching 100% in both its acute and peracute forms (1). First documented in Kenya in 1921, ASF has been reported in over 83 countries or regions across Africa, Europe, Asia, Oceania, and the Americas (WOAH, https://www.woah.org/en/disease/african-swine-fever/). In 2018, outbreaks worsened considerably when ASFV was detected in China, constituting over half of the global pig population (2, 3). The ongoing worldwide spread of ASF poses a critical threat to global pork production and food security, causing a substantial socioeconomic burden. Although two deletion mutant attenuated live vaccines (ASFV-G-ΔI177L and ASFV-G-ΔMGF) have been licensed in Vietnam, their general applicability in ASF management remains to be demonstrated (4). Existing control options are limited to culling and biosecurity measures, highlighting the urgent need for safe and effective vaccines and targeted therapeutics.

ASFV is a large, complex, cytoplasmic DNA virus constituting the sole member of the Asfarviridae family (5). The viral genome consists of a linear double-stranded DNA molecule of 170–194 kbp that encodes up to 170–190 open reading frames (ORFs), allowing viral genome replication, transcription, translation, morphogenesis/assembly, and immune escape (6, 7). Genomic variation between strains is predominantly due to the gain or loss of multigene family members (MGFs). Many of these genes code for proteins that are nonessential for replication in macrophages *in vitro* but inhibit host defenses and are, therefore, associated with virulence in domestic pigs (8–15). The deletion of multiple MGF genes provides an attractive route for producing rationally attenuated vaccine candidates (4). Nevertheless, our limited understanding of the complex pathogenesis of ASFV and its diverse immune evasion strategies remains a major obstacle to developing vaccines and therapeutics.

The integrated stress response (ISR) is a conserved eukaryotic stress response network that, upon activation by various stress stimuli, profoundly reprograms translation and restores cellular homeostasis. It is coordinated by at least four stress-responsive kinases, namely PKR-like ER kinase (PERK), double-stranded RNA-dependent protein kinase (PKR), heme-regulated eIF2α kinase (HRI), and general control nonderepressible 2 (GCN2), the action of all four of which converges on phosphorylation of the α-subunit of eukaryotic translation initiation factor 2 (eIF2α) on serine (S51). This leads to a reduction in global protein synthesis while allowing the preferential translation of ISR-specific mRNAs, including activating transcription factor 4 (ATF4), that together direct cell survival and recovery. Although a short-lived ISR is primarily a pro-survival, homeostatic program, prolonged ISR can drive signaling toward cell death induction. Therefore, the regulation of eIF2α phosphorylation is a central mechanism determining the balance of cell death and survival by directing translational reprogramming and, ultimately, the cellular outcome of the ISR (16–18). As intracellular parasites, viruses hijack host cell protein synthesis machinery to synthesize viral proteins and package new viral particles, which are inevitably patrolled by a multilayered intracellular immune response (19–21). The ISR is a prominent antiviral pathway that causes global translational arrest, resulting in virion production inhibition. However, viruses have evolved diverse strategies to evade immune activation and its consequences. The regulation of the ISR and eIF2α phosphorylation has been regarded as a critical step for viral replication, with remarkable influences on virulence, tissue tropism, and pathogenicity (22, 23). Therefore, elucidating the virus–host interactions involved in these processes provides invaluable implications for developing novel vaccines and/or antiviral therapies.

The endoplasmic reticulum (ER) is an essential organelle for ASFV replication and maturation (24, 25). ASFV morphogenesis occurs at perinuclear ER-derived membranes termed viral factories (VFs), and viral structural proteins such as p54 and pE146L have been implicated in recruiting or remodeling ER membranes for virion envelopment (26–29). Accumulating evidence from recent studies using wild-type ASFV strains in physiologically relevant primary macrophages and *in vivo* pig models has demonstrated that ASFV infection robustly triggers ER stress and activates the unfolded protein response (UPR) to facilitate viral replication (30–32). Several viral proteins critically influencing ER structure and function have also been identified. For instance, ASFV pEP152R, an early ER-localized membrane protein, robustly induces ER stress and swelling, triggering the PERK/eIF2α pathway and potently inhibiting host protein synthesis (33). The transmembrane (TM) protein pD117L interacts with multiple UPR-associated proteins and directly triggers UPR activation (32). Another transmembrane protein, pE146L, triggers perinuclear aggregation of the ER and has been implicated in supplying membrane material for viral morphogenesis (29). Additionally, ectopic expression of pE66L or pMGF110-7L has been shown to induce eIF2α phosphorylation, the primary activator of the ISR, leading to the shutdown of global protein synthesis (34, 35). In contrast, the late viral protein DP71L recruits protein phosphatase 1 (PP1) to dephosphorylate eIF2α and suppress certain aspects of the UPR, particularly the PERK-eIF2α-CHOP axis (36, 37). The complex interactions between virus-encoded functions, ER remodeling, and the ISR play key roles in determining the outcome of ASFV infection; however, they remain poorly understood.

In this study, we comprehensively analyzed the relationship between viral proteins and ISR regulation. We found that the ASFV virulence-related factor pMGF110-9L was the strongest activator of *ATF4* gene expression—the main effector of the ISR. A single deletion of MGF110-9L significantly decreased the ability of viral replication *in vitro* and partially weakened virulence *in vivo* (13), and the deletion of MGF110-9L together with MGF505-7R or MGF360-9L markedly reduced virulence and conferred protection against homologous challenge (14, 38). MGF110-9L suppresses type I interferon production by mediating the autophagic degradation of TBK1 (15, 39). In the present study, we revealed a previously uncharacterized role of pMGF110-9L in the induction of eIF2α phosphorylation and ISR signaling, resulting in global protein synthesis arrest and stress granule (SG) formation, in the context of both exogenous expression and ASFV infection. Further mechanistic investigations demonstrated that pMGF110-9L-induced ISR predominantly results from ER stress induction. This study advances our understanding of the pathogenic mechanisms of ASFV and highlights the involvement of pMGF110-9L in ASFV infection and pathogenesis.

## RESULTS

### Luciferase-based genome-wide screening of ASFV-encoded ISR modulators

**Fig 1:**
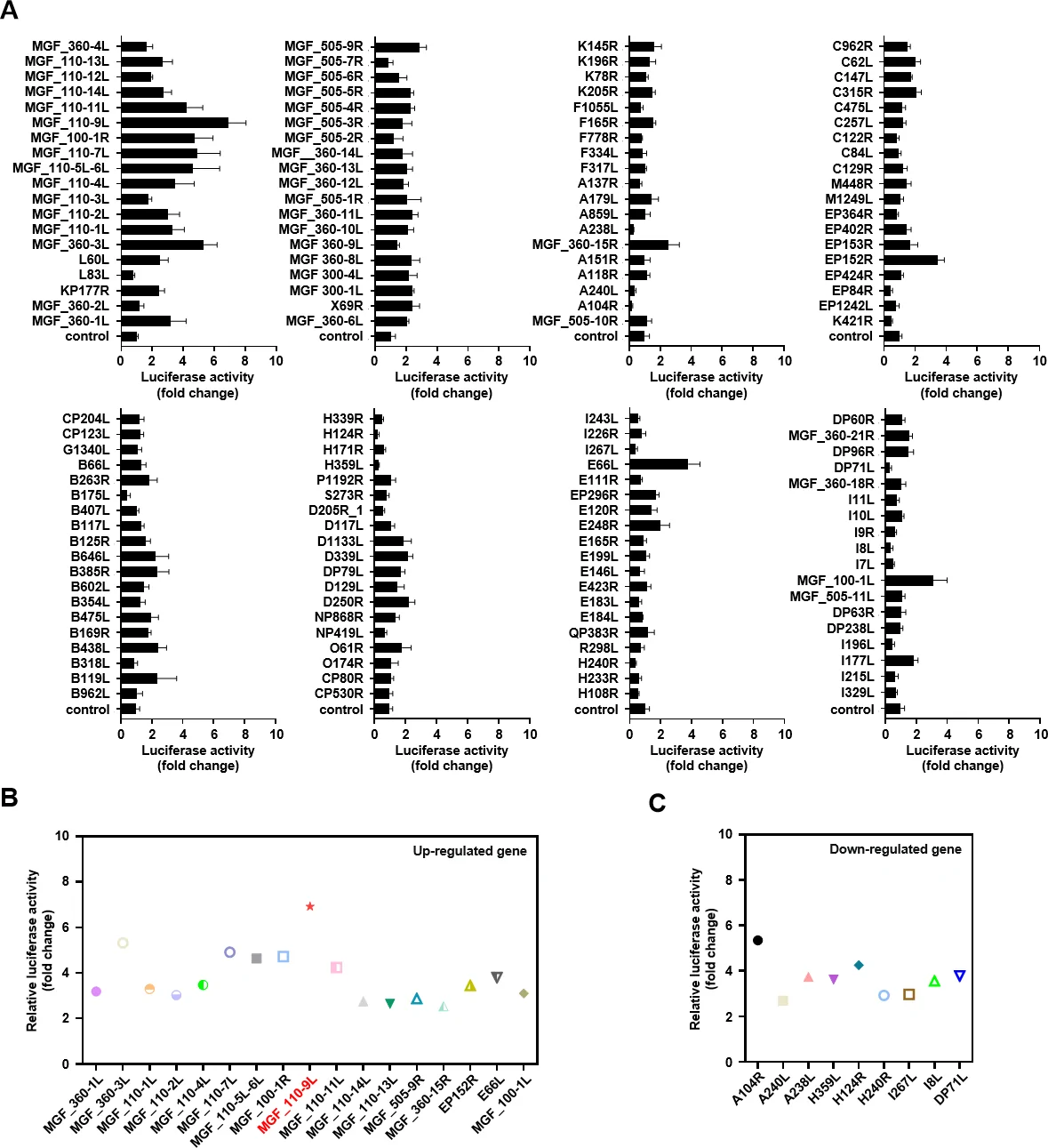
Preliminary screening of ASFV genes for modulation of ATF4-Rluc gene expression. (**A**) HEK-293T cells were cotransfected with plasmids of individual ASFV cDNA (0.1 µg) and ATF4-Rluc reporter (0.05 µg). At 24 h post-transfection, the cells were lysed and subjected to the Rluc assay. (**B**) Seventeen ASFV proteins that stimulate ATF4-Rluc activity at levels greater than 2.5-fold were screened, with pMGF110-9L having the most significant effect. (**C**) Nine ASFV proteins that inhibited ATF4-Rluc gene expression by more than 2.5-fold were screened. Data are the means of three independent experiments, and error bars indicate standard deviations (SD).

Phosphorylation of eIF2α is the major mechanism underlying the ISR and significantly stimulates the preferential translation of ATF4 (16, 17). To screen for ASFV-encoded ISR regulators on a genome-wide scale, an ATF4 luciferase (ATF4-Rluc) reporter system was used to detect ATF4 activity in human embryonic kidney 293T (HEK293T) cells ectopically expressing 151 ORFs of the highly virulent ASFV China/2018/AnhuiXCGQ strain (34). The results indicated that multiple ASFV-encoded proteins affect the expression of ATF4-Rluc to varying degrees (Fig. 1A), suggesting that the overall biological effects observed during viral infection may arise from integrated regulatory mechanisms. To eliminate the possibility that the reduced luciferase signals were due to impaired cell viability, we performed Cell Counting Kit-8 (CCK-8) assays, which confirmed that exogenous expression of the tested ASFV proteins showed no obvious cytotoxicity or growth-inhibitory effects (Fig. S1).

To identify ASFV proteins that play the most dominant roles in host ISR activation, we established that the increase in ATF4-Rluc expression must be >2.5-fold higher than that of a mock control. Based on this criterion, we found that 17 proteins robustly increased the expression of ATF4-Rluc, including most members of the pMGF110 family, multiple members of the pMGF360 family, pMGF100-1L, pMGF100-1R, pMGF505-9R, pE66L, and pEP152R (Fig. 1B). Most of these proteins are expressed during the early phases of viral infection (40). In addition, we identified ASFV proteins that markedly inhibited ATF4-Rluc activity by >2.5-fold, including pA104R, pA240L, pA238L, pH359L, pH124R, pH240R, pI267L, pI8L, and pDP71L. In contrast to the positive regulators, most of these genes are expressed during the late stages of infection (40) (Fig. 1C). To eliminate the possibility that the observed differences in ATF4-Rluc activity were due to variations in protein expression levels, we examined the expression of the screened ASFV proteins by indirect immunofluorescence assay. The results confirmed that all proteins were efficiently expressed at comparable levels in transfected cells (Fig. S2 and S3). Consistent with our screening results, previous studies have revealed that the two membrane proteins pE66L and pEP152R trigger the phosphorylation of eIF2α (33, 35), whereas pDP71L dephosphorylates eIF2α and inhibits ATF4 induction (41). This supports the validity of our experimental approach.

Taken together, these findings provide a more comprehensive understanding of how individual ASFV proteins influence the host ISR and establish their importance in viral replication and virulence. Notably, we identified pMGF110-9L as the strongest stimulatory factor for ATF4-Rluc expression (Fig. 1A and B). Previously, we demonstrated by deletion experiments that MGF110-9L is a potent virulence-associated gene with potential as a promising target for modified live virus (MLV) vaccines (13, 14, 38). However, the mechanism of the effects of MGF110-9L deletion remains unclear, highlighting the need for further investigation into the potential role of pMGF110-9L in mediating the ISR.

### ASFV pMGF110-9L robustly induces the phosphorylation of eIF2α and activates the ISR

**Fig 2:**
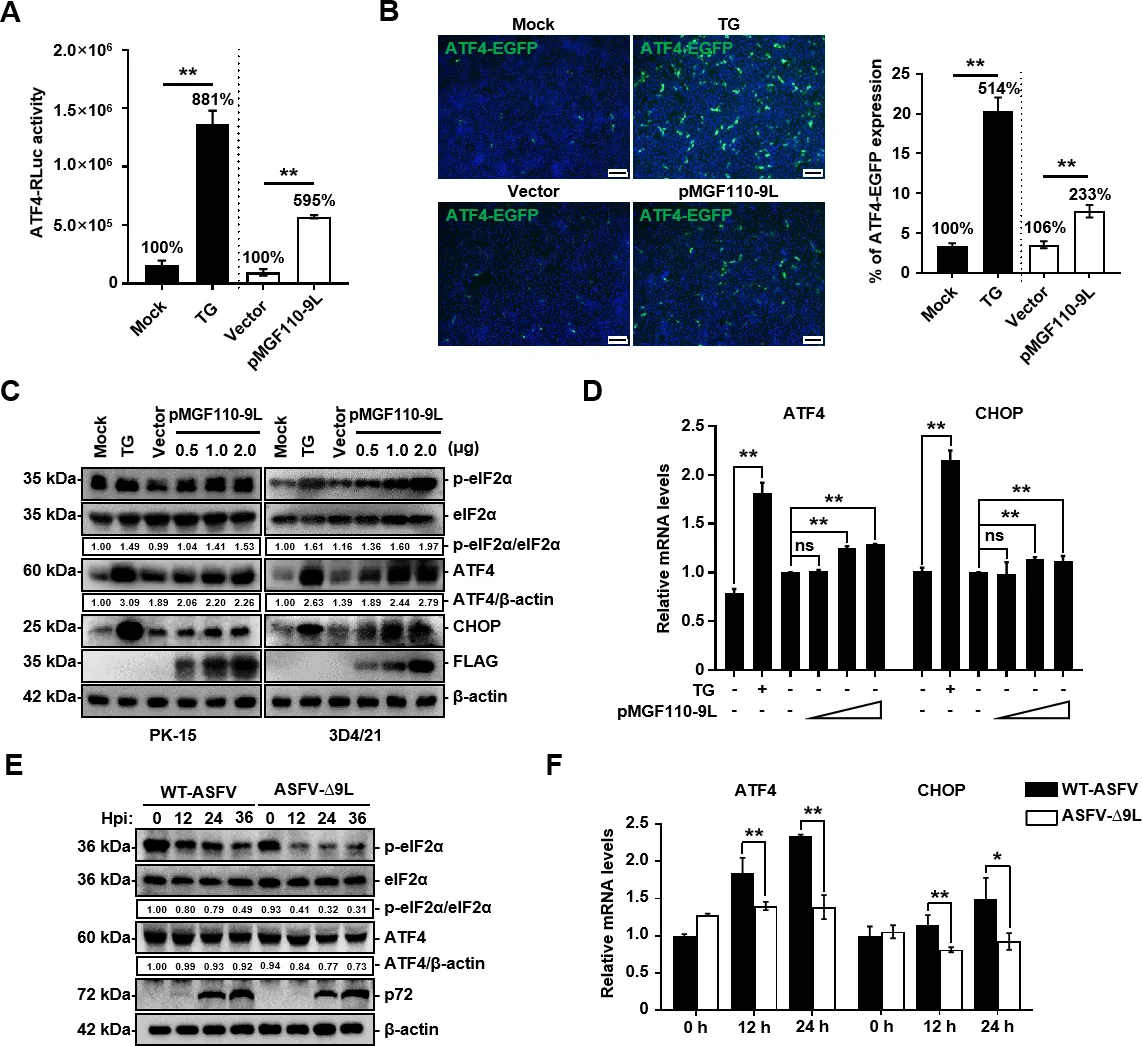
ASFV pMGF110-9L induces the phosphorylation of eIF2α and activates the ISR. (**A**) PK-15 cells were cotransfected with the ATF4-RLuc reporter (0.05 µg) and the pMGF110-9L expression vector or an empty vector (0.1 µg), respectively. A positive control for eIF2α phosphorylation was set by TG (1 µM) treatment for 6 h on cells transfected with ATF4-RLuc. At 24 h post-transfection, cells were lysed and subjected to the Rluc assay. (**B**) PK-15 cells were cotransfected with the ATF4-EGFP reporter (0.1 µg) and the pMGF110-9L expression vector or an empty vector (0.2 µg), respectively. TG was used as the positive control. At 24 h post-transfection, the cells were subjected to fluorescence analysis. Scale bar, 100 µm. The right panel shows the percentage of cells expressing EGFP in each group under different treatments. (**C**) PK-15 and 3D4/21 cells were transfected with the pMGF110-9L expression vector at increasing doses (0.5, 1.0, and 2.0 µg) or with an empty vector (2.0 µg) for 24 h. TG was used as the positive control. The whole-cell lysates were subjected to Western blot analysis. (**D**) 3D4/21 cells were treated as described for panel C. RNA samples were extracted, and the mRNA levels of ATF4 and CHOP were then detected by RT-qPCR analysis. (**E and F**) PAMs were uninfected or infected with ASFV-WT or ASFV-Δ9L (multiplicity of infection [MOI] = 0.1). At the indicated times, the whole-cell lysates were subjected to Western blot analysis with the indicated antibodies, or RT-qPCR analysis for ATF4 and CHOP mRNA levels. Data are presented as the mean ± SD of three independent experiments. *, *P* < 0.05; **, *P* < 0.01; ns, not significant.

To confirm whether pMGF110-9L functions as a potent inducer of ATF4 translation, ATF4-Rluc and ATF4-EGFP reporters were used to evaluate ATF4 activity in porcine kidney-15 (PK-15) cells ectopically expressing pMGF110-9L or treated with the positive control thapsigargin (TG), a well-characterized ER stress- and ISR-inducing agent (42). As expected, we observed a significant increase in both the activity of ATF4-Rluc and the fluorescence signal of ATF4-EGFP in TG-treated cells compared with those in untreated cells. Consistent with the screening results, ectopic expression of pMGF110-9L also significantly increased the specific ATF4-Rluc activity (~6.0-fold) and ATF4-EGFP signal (~2.3-fold) (Fig. 2A and B). Furthermore, a CCK-8 assay revealed that the ectopic expression of pMGF110-9L at the indicated doses had a negligible effect on cell viability (Fig. S4).

To determine whether pMGF110-9L plays an important role in the induction of eIF2α phosphorylation and ISR activation, we performed immunoblotting to evaluate the levels of phosphorylated eIF2α (p-eIF2α) and ATF4 protein in PK-15 cells and porcine alveolar macrophage-derived 3D4/21 cells ectopically expressing a concentration range of pMGF110-9L. As shown in Fig. 2C, a significant increase in the levels of p-eIF2α and ATF4 protein was observed following TG treatment, and ectopic expression of pMGF110-9L also remarkably increased eIF2α phosphorylation and ATF4 expression in a dose-dependent manner in both cell lines. Furthermore, quantitative real-time RT-PCR (RT-qPCR) analysis was conducted to detect the transcript levels of key ISR regulatory genes. Consistently, the results showed that the mRNA levels of ATF4 and CHOP significantly increased in response to increased expression of pMGF110-9L (Fig. 2D).

To further investigate the involvement of pMGF110-9L in eIF2α signaling and ISR during ASFV infection, we compared the levels of p-eIF2α and ATF4 protein in primary porcine alveolar macrophages (PAMs) infected by MGF110-9L-deficient ASFV (designated as ASFV-Δ9L) and its parental ASFV CN/GS/2018 (ASFV-WT). P-eIF2α levels were reduced in the context of both ASFV-WT and ASFV-Δ9L infection from 12 h post-infection (hpi) onward, remaining suppressed through at least 36 hpi (Fig. 2E; Fig. S5), which is consistent with previous reports (32, 43, 44). Notably, we observed a stronger decrease in the levels of p-eIF2α in PAMs infected with ASFV-Δ9L compared with ASFV-WT, which correlated with a stronger decrease of ATF4 protein level (Fig. 2E; Fig. S5). Consistently, qPCR analysis indicated that depletion of MGF110-9L in the parental strain led to a significant decrease in the mRNA levels of ATF4 and CHOP (Fig. 2F). Taken together, these findings indicate that pMGF110-9L contributes to the induction of eIF2α phosphorylation and ISR signaling, as well as subsequent programs of stress-induced gene expression.

### ASFV pMGF110-9L induces ER swelling and an abnormal ER morphology

**Fig 3:**
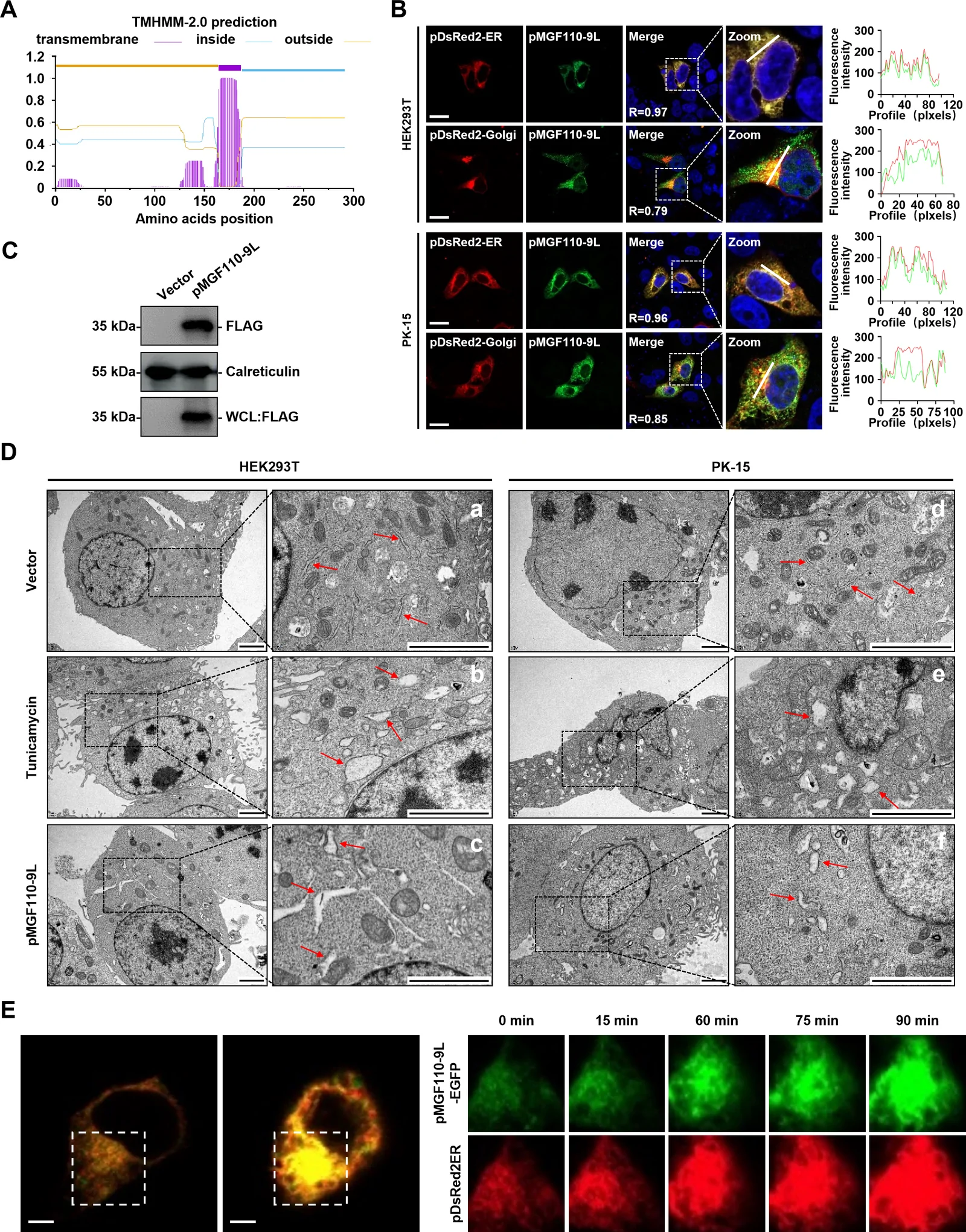
ASFV pMGF110-9L induces ER swelling and an abnormal ER morphology. (**A**) Predicted transmembrane domains in pMGF110-9L through TMHMM-2.0 analysis. (**B**) HEK293T and PK-15 cells were cotransfected with the pMGF110-9L expression vector (0.5 µg) and one of the following organelle markers: pDsRed2-ER or pDsRed2-Golgi (0.2 µg, red). At 24 h post-transfection, the cells were fixed, permeabilized, and incubated with an anti-FLAG antibody, followed by incubation with an AF488-conjugated secondary antibody (green). Nuclei were counterstained with DAPI (blue). Cells were imaged using confocal microscopy. Scale bar, 20 µm. The co-localization coefficient was statistically analyzed using ImageJ. (**C**) PK-15 cells transfected with the pMGF110-9L expression vector or an empty vector were analyzed for ER isolation and Western blotting at 24 h post-transfection. (**D**) Transmission electron microscopy (TEM) revealed ER morphology in HEK293T and PK-15 cells ectopically expressing pMGF110-9L. Scale bar: 2 µm as indicated. (**E**) SIM imaging tracked dynamic changes of pMGF110-9L-EGFP (green) and pDsRed2-ER (red) in HEK293T cells. Scale bar, 5 µm.

MGF110-9L is highly conserved among ASFV strains and is transcribed during the early stages of infection (13). Bioinformatics analysis revealed that pMGF110-9L contains a signal peptide domain and a highly conserved cysteine-rich domain [C-(X)5-C-(X)2-C-(X)2-C-(X)4-C] at the N-terminus (Fig. S6 and S7), suggesting its involvement in the secretory pathway and function in the oxidizing environment of the ER lumen. pMGF110-9L was predicted to possess a 23-amino-acid TM domain, with the N-terminus projecting into the organelle lumen (Fig. 3A). To accurately define the specific organelle membrane housing pMGF110-9L, we co-transfected HEK293T and PK-15 cells with a vector expressing pMGF110-9L and an organelle-specific marker encoding a fusion of DsRed2 with the targeting sequence of the corresponding organelle. Fluorescence imaging and subsequent colocalization analysis revealed that pMGF110-9L exhibited predominant colocalization with the ER and partial co-localization with the Golgi apparatus, but no co-localization with the mitochondria, late endosome/lysosome, or peroxisome (Fig. 3B; Fig. S8). Furthermore, using an ER isolation kit, we demonstrated a significant enrichment of over-expressed pMGF110-9L in the ER fractions (Fig. 3C). Strikingly, ectopic expression of pMGF110-9L produced unusual perinuclear structures that contained resident ER proteins as well as triggered a significant reorganization of the morphological characteristics of the Golgi membrane network (Fig. 3B). This suggests that pMGF110-9L is an ER-localized membrane protein that functions in the redistribution of resident ER proteins and aberrant trafficking of resident Golgi proteins from the ER.

To confirm this phenomenon of induced profound rearrangement, transmission electron microscopy (TEM) was used to observe ER morphology in HEK293T and PK-15 cells ectopically expressing pMGF110-9L. This revealed a swollen ER and increased ER volume in response to pMGF110-9L expression and TG treatment in both cell lines (Fig. 3D). We also performed time-lapse imaging with structured illumination microscopy (SIM) to capture the dynamic nature of pMGF110-9L-induced ER rearrangement in HEK293T cells. pMGF110-9L expression induced the gradual transition of resident ER proteins from elongated strips to clustered aggregates over time, which correlated with a profound perinuclear accumulation of pMGF110-9L (Fig. 3E). Collectively, these results indicate that pMGF110-9L robustly induces ER swelling and abnormal ER morphology, potentially causing stress and exerting a critical effect on ER homeostasis.

### ASFV pMGF110-9L induces ER stress and triggers the activation of three UPR pathways

To characterize the potential mechanism of pMGF110-9L-induced ISR, a comparative proteomic analysis of pMGF110-9L-overexpressing cells was performed to reveal significant alterations in the host proteome (Fig. S9A). Gene Ontology enrichment analysis revealed significant changes in translation regulation, unfolded protein binding, and coated vesicles (Fig. S9B). STRING network analysis linked pMGF110-9L to protein processing in the ER, ER membrane protein complex, calcium signaling pathway, and ATP metabolism (Fig. S9C). KEGG pathway enrichment analysis further suggested the involvement of pMGF110-9L in the regulation of protein processing in the ER and metabolic pathways related to cellular energy (Fig. S9D). Combined with the unusual localization and abnormal ER morphology, it was speculated that pMGF110-9L-mediated ISR could be triggered by ER stress, an intrinsic cellular stress resulting from the accumulation of unfolded and misfolded proteins in the ER and ER swelling.

**Fig 4:**
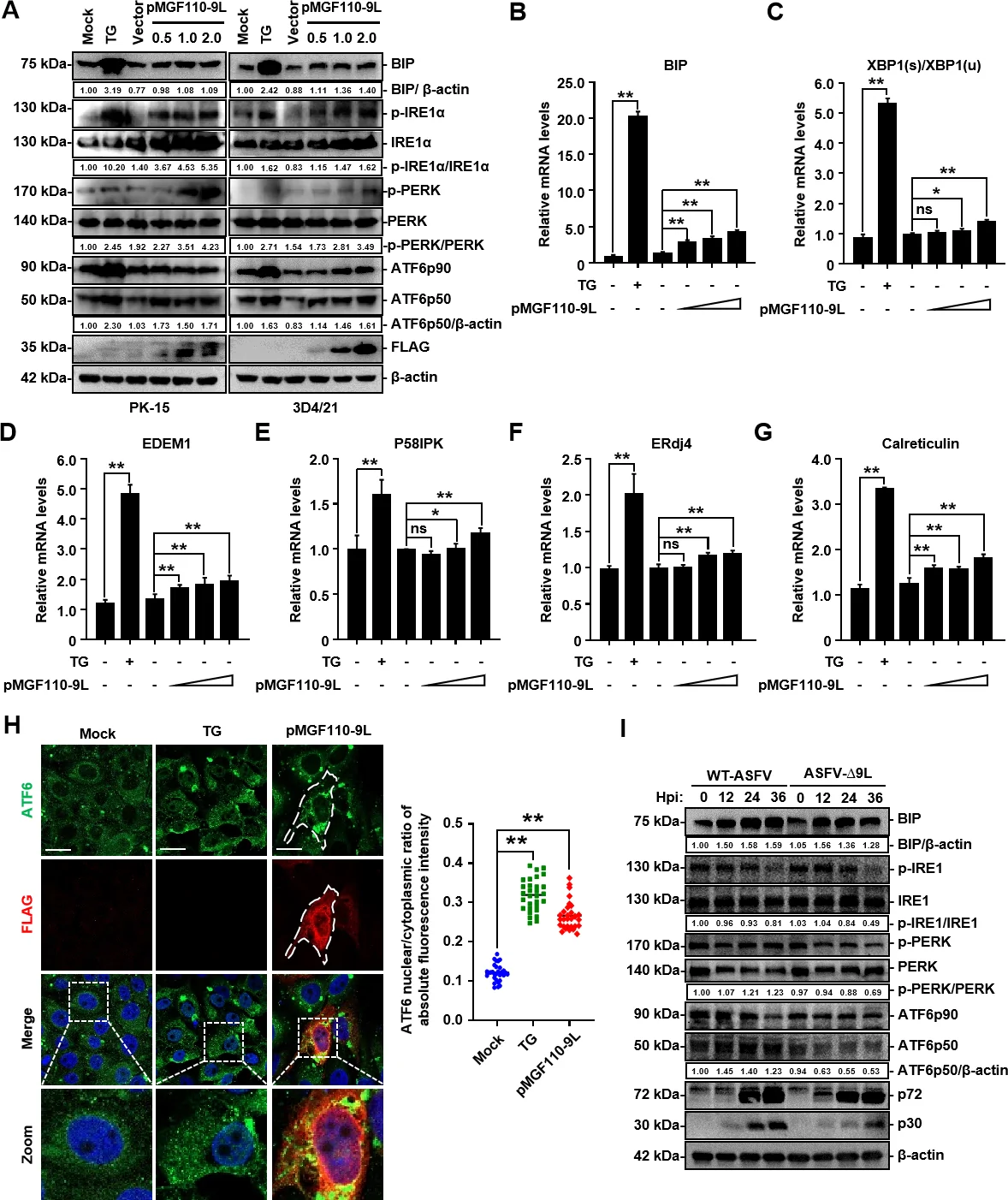
ASFV pMGF110-9L induces ER stress and activates the three branches of the UPR. (**A**) PK-15 and 3D4/21 cells were transfected with the pMGF110-9L expression vector at increasing doses (0.5, 1.0, and 2.0 µg) or with an empty vector (2.0 µg) for 24 h. TG was used as the positive control. The whole-cell lysates were subjected to Western blot analysis. (B–G) 3D4/21 cells were treated as described for panel A. RNA samples were extracted, and the mRNA levels of BIP (**B**), XBP1(s)/XBP1(u) (**C**), EDEM1 (**D**), P58IPK (**E**), ERdj4 (**F**), and Calreticulin (**G**) were then determined by RT-qPCR analysis. (**H**) Representative immunofluorescence images (left) of PK-15 cells transfected with the pMGF110-9L expression vector (0.5 µg) for 24 h or treated with TG (1 µM) for 6 h, followed by incubation with anti-ATF6 (green) and anti-FLAG (red) antibodies. Nuclei were stained with DAPI (blue). Scale bar, 20 µm. The right panel shows the nuclear-to-cytoplasmic fluorescence intensity ratio of ATF6, quantified from ≥30 cells per condition across three independent experiments. (**I**) PAM cells were infected with ASFV-WT or ASFV-Δ9L (MOI = 0.1). At the indicated times, the whole-cell lysates were subjected to Western blot analysis with the indicated antibodies. Data are presented as the mean ± SD of three independent experiments. *, *P* < 0.05; **, *P* < 0.01; ns, not significant.

To functionally validate the protein interactions between pMGF110-9L and host ER-resident chaperones, three ER homeostasis regulators—BIP, calreticulin, and protein disulfide-isomerase (PDI)—were selected based on their identification in our proteomic analysis. Reciprocal co-immunoprecipitation assays confirmed robust physical interactions between pMGF110-9L and all three chaperones (Fig. S10A through C). Double-label immunofluorescence further demonstrated significant colocalization of pMGF110-9L with BIP, calreticulin, and PDI within the ER compartment (Fig. S10D through F). To further determine whether pMGF110-9L alone is sufficient to induce ER stress, we analyzed the expression level of the major ER stress marker BIP in pMGF110-9L-overexpressing cells, again using the ER stress activator TG as a positive control. Notably, we observed that both the mRNA and protein levels of BIP gradually increased in a dose-dependent manner with respect to pMGF110-9L overexpression in both PK-15 and 3D4/21 cells (Fig. 4A and B), confirming the function of pMGF110-9L in triggering ER stress.

The regulation of ER homeostasis relies on the activation of three ER transmembrane sensors, namely PERK, inositol-requiring enzyme 1α (IRE1α), and ATF6 of the UPR pathway (45, 46). Therefore, we analyzed the transcription and protein expression of key molecules in the three branches of the UPR pathway. Immunoblotting results revealed that ectopic expression of pMGF110-9L significantly enhanced the phosphorylation level of PERK (P-PERK) and IRE1α (P-IRE1α) and increased the abundance of the 50-kDa active variant of ATF6 (ATF6p50), in a dose-dependent manner (Fig. 4A). Consistently, RT-qPCR demonstrated that the mRNA levels of spliced Xbp1s and its downstream genes EDEM1, P58IPK, ERdj4, and the ATF6p50-targeted gene calreticulin were also significantly elevated in pMGF110-9L-expressing 3D4/21 cells (Fig. 4C through G). Confocal immunofluorescence revealed stronger nuclear signals for ATF6p50 in pMGF110-9L-expressing and TG-treated PK-15 cells than those in the control group (Fig. 4H). Furthermore, we investigated the effects of pMGF110-9L on the activation of ER stress and UPR during ASFV infection. Immunoblotting results revealed a progressive increase in the expression levels of BIP and activated ATF6p50, but not p-PERK and p-IRE1α, in ASFV-WT-infected PAMs (Fig. 4I; Fig. S11), consistent with a previous study (31). Notably, depletion of MGF110-9L led to a strong decrease in the level of BIP, p-PERK, p-IRE1α, and activated ATF6p50 (Fig. 4I; Fig. S11), suggesting that pMGF110-9L is a major factor driving ASFV-induced UPR. Taken together, our findings demonstrate that pMGF110-9L is crucial for ASFV-induced ER stress, leading to the activation of the PERK-eIF2α-ATF4, IRE1α-XBP1, and ATF6 branches of the UPR.

### ASFV pMGF110-9L activates the ISR through the PERK/PKR-eIF2α pathway

**Fig 5:**
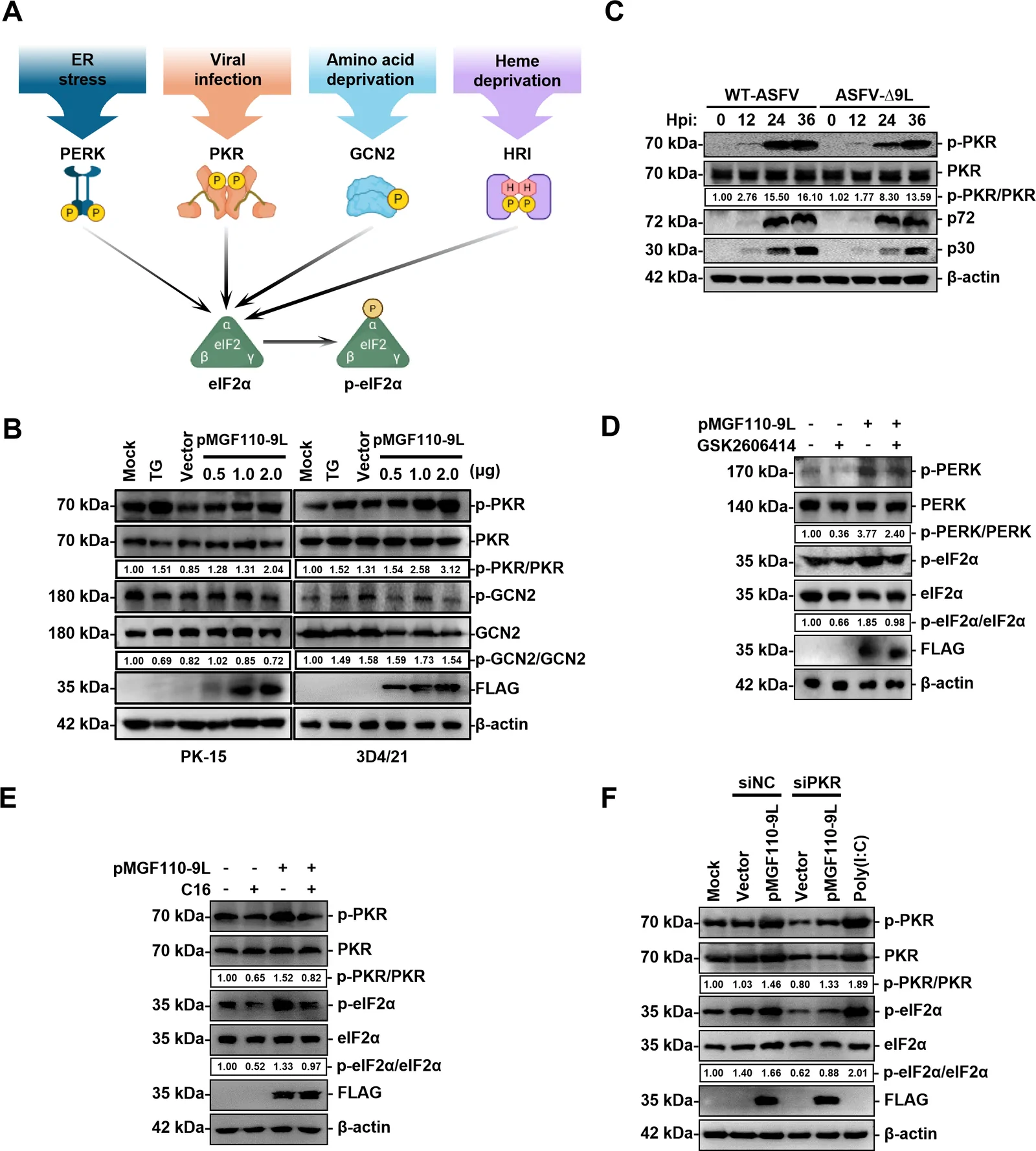
ASFV pMGF110-9L enhances eIF2α phosphorylation by upregulating PERK and PKR activity. (**A**) Diagrams of four major stress pathways leading to eIF2α phosphorylation by different kinases, PERK, PKR, GCN2, and HRI. (**B**) PK-15 and 3D4/21 cells were transfected with the pMGF110-9L expression vector at increasing doses (0.5, 1.0, and 2.0 µg) or with an empty vector (2.0 µg) for 24 h. TG was used as the positive control. The whole-cell lysates were subjected to Western blot analysis for examination of the phosphorylation and expression of PKR and GCN2. (**C**) PAM cells infected with ASFV-WT or ASFV-Δ9L (MOI = 0.1) for the indicated times were lysed and subjected to Western blot analysis with the indicated antibodies. (**D and E**) 3D4/21 cells transfected with the pMGF110-9L expression vector (2.0 µg) or with an empty vector were treated with GSK2606414 (10 µM) or C16 (1 µM). After 24 h post-transfection, the whole-cell lysates were subjected to Western blot analysis for examination of the phosphorylation and expression of PERK, PKR, and eIF2α. (**F**) 3D4/21 cells were transfected with negative-control siRNA (siNC) or PKR-specific siRNA (siPKR) for 30 h, followed by transfection with an empty vector or a pMGF110-9L expression plasmid. After 24 h post-transfection, the whole-cell lysates were subjected to Western blot analysis for examination of the phosphorylation and expression of PKR and eIF2α.

The common point of convergence for diverse stresses that activate the ISR is eIF2α phosphorylation, which can be coordinated by any of the four cellular kinases PERK, PKR, GCN2, or HRI (Fig. 5A). Our results demonstrated that the ectopic expression of pMGF110-9L can activate the PERK-eIF2α-ATF4 pathway (Fig. 2C and 4A). To further define the mechanism of pMGF110-9L-induced eIF2α phosphorylation, we investigated the expression and phosphorylation of the kinases PKR and GCN2 in PK-15 and 3D4/21 cells ectopically expressing pMGF110-9L. Strikingly, we observed a significant and progressive increase in the phosphorylation of PKR (p-PKR), but not GCN2 (p-GCN2), in response to increasing expression of pMGF110-9L (Fig. 5B). Notably, pMGF110-9L-induced PKR activation was more pronounced in 3D4/21 cells than in PK-15 cells (Fig. 5B). This difference likely reflects the fact that 3D4/21 cells are porcine alveolar macrophages with intrinsic innate immune functions, which may exhibit higher basal PKR expression and more robust activation of dsRNA-dependent signaling and interferon-related pathways compared to PK-15 epithelial cells (47). To clarify the effect of pMGF110-9L on the activation of PKR during ASFV infection, we compared the levels of p-PKR in PAMs infected with ASFV-WT and ASFV-Δ9L. Consistently, we observed an extremely significant increase in p-PKR levels in PAMs with the progression of infection with both forms of ASFV, but a stronger decrease in the p-PKR level was observed in ASFV-Δ9L-infected PAMs compared with ASFV-WT (Fig. 5C; Fig. S12), indicating that PKR also plays a critical role in pMGF110-9L-induced eIF2α phosphorylation.

To further validate the critical roles of PERK and PKR in pMGF110-9L-mediated eIF2α phosphorylation, specific inhibitors targeting PERK (GSK2606414) and PKR (C16) were used to treat pMGF110-9L-expressing or control 3D4/21 cells. Immunoblotting results revealed that the PERK/PKR-eIF2α pathway was markedly inhibited upon treatment with GSK2606414 or C16, and pMGF110-9L-induced phosphorylation of PERK, PKR, and eIF2α was substantially decreased upon treatment with GSK2606414 or C16 (Fig. 5D and E). Considering that C16 is a non-selective PKR inhibitor with potential off-target effects, we further performed PKR-specific siRNA knockdown experiments to obtain genetic evidence independent of pharmacological inhibition. Knockdown of endogenous PKR drastically suppressed pMGF110-9L-induced phosphorylation of PKR and eIF2α, consistent with the results obtained from C16 treatment (Fig. 5F). Collectively, these data demonstrate that pMGF110-9L induces the phosphorylation of eIF2α and activates the ISR by activating the PERK and PKR pathways.

### ASFV pMGF110-9L inhibits cellular translation in an eIF2α phosphorylation-dependent manner

**Fig 6:**
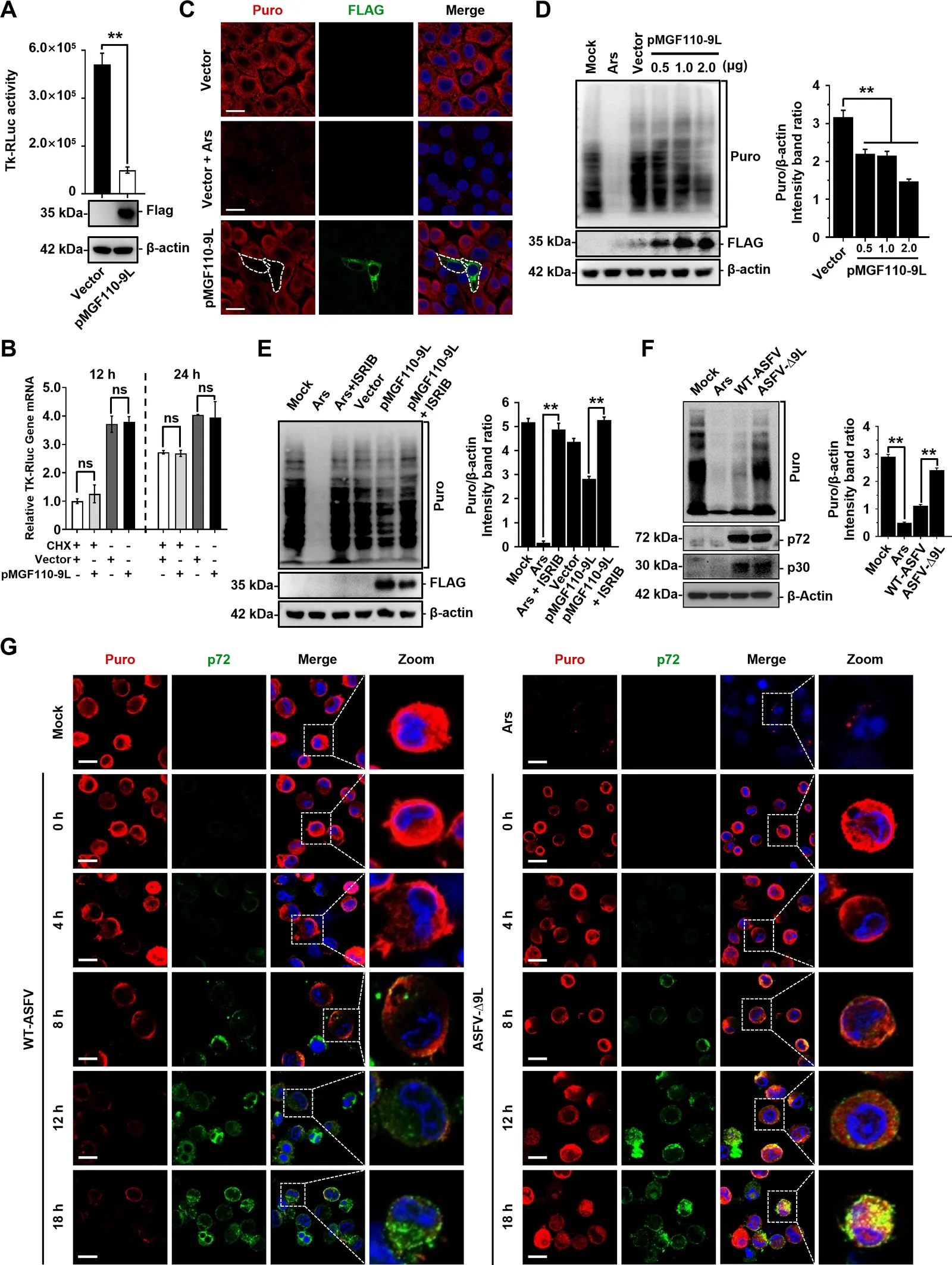
ASFV pMGF110-9L has a broad inhibitory effect on host cell protein synthesis. (**A**) HEK293T cells were cotransfected with pRL-TK (0.1 µg) and pMGF110-9L expression vector or an empty vector (0.1 µg), respectively. At 24 h post-transfection, the cells were lysed and subjected to the Rluc assay or Western blot analysis. (**B**) The effect of pMGF110-9L overexpression on Rluc mRNA production was examined by qRT-PCR when the cells were pretreated with or without CHX at 12 and 24 h. (**C**) PK-15 cells transfected with the pMGF110-9L expression vector or with an empty vector (0.5 µg) for 24 h were labeled with puromycin (5 μg/mL) for 45 min. As a positive control, cells were treated with 0.5 mM arsenite (Ars) for 45 min before the addition of puromycin. The cells were then fixed, permeabilized, and processed for immunofluorescence assay. A FLAG-specific monoclonal antibody was used to detect pMGF110-9L (green). Puromycylated chains were visualized using an anti-puromycin antibody (red). Nuclei were stained with DAPI (blue). Scale bar, 20 µm. (**D**) PK-15 cells were transfected with the pMGF110-9L expression vector at increasing doses (0.5, 1.0, and 2.0 µg) or with an empty vector (2.0 µg). After 24 h, the cells were labeled with puromycin for 45 min. The whole-cell lysates were subjected to Western blot analysis. Densitometric signal targeting puromycin was determined by ImageJ (right). (**E**) 3D4/21 cells treated with 0.5 mM Ars for 45 min or transfected with the pMGF110-9L expression vector for 24 h were incubated with or without ISRIB (0.5 µM). Thirty minutes before the cell lysates were harvested, the cells were labeled with puromycin and immunolabeled with anti-puromycin antibody to visualize the nascent polypeptide chains. (**F**) PAM cells were infected with ASFV-WT or ASFV-Δ9L (MOI = 0.1). At 18 h post-infection, cells were labeled with puromycin and then subjected to Western blot analysis. Treatment with Ars served as a positive control. (**G**) PAM cells infected with ASFV-WT or ASFV-Δ9L for the indicated times were labeled with puromycin and then proceeded to immunofluorescence assay. An anti-p72 polyclonal antibody was used to detect ASFV protein (green). Puromycylated chains were visualized using an anti-puromycin antibody (red). Nuclei were stained with DAPI (blue). Scale bar, 20 µm. Data are presented as the mean ± SD of three independent experiments. **, *P* < 0.01; ns, not significant.

A major consequence of eIF2α phosphorylation and ISR activation is the global reduction of host cell protein synthesis (16, 17). Therefore, we performed an Rluc assay with a nonspecific Renilla gene to examine the effect of pMGF110-9L on host gene expression in HEK293T cells. The results revealed that Rluc expression in pMGF110-9L-expressing cells was significantly lower than that in the control cells (Fig. 6A). To further investigate the step at which pMGF110-9L affects, qRT-PCR analysis was performed with cycloheximide (CHX)-treated cells. The levels of Rluc mRNA were observed to be comparable between pMGF110-9L-overexpressing cells and control cells, both in the presence and absence of CHX from 12 to 24 h (Fig. 6B). This suggested that pMGF110-9L downregulates Rluc expression at the translational level. To determine whether pMGF110-9L affects cellular protein synthesis, we investigated global translational efficiency using a ribopuromycylation assay (48). As a positive control, treatment with sodium arsenite (Ars), a potent inducer of eIF2α phosphorylation (49), caused a profound absence of puromycin incorporation (Fig. 6C through F). Notably, we also observed a significant and dose-dependent reduction in global protein synthesis in PK-15 cells overexpressing pMGF110-9L (Fig. 6C and D). To further address whether pMGF110-9L-induced ISR is directly related to global host translation arrest, we used ISRIB, a known pharmacologic ISR inhibitor that can reverse the inhibitory impact of eIF2α phosphorylation (50). Immunoblotting revealed that ISRIB treatment of pMGF110-9L-expressing cells significantly restored global translational efficiency compared to untreated cells (Fig. 6E). Furthermore, we compared the rate of global cellular protein synthesis in PAMs infected by ASFV-WT and ASFV-Δ9L using the ribopuromycylation assays. The results showed that cellular protein synthesis was significantly downregulated in ASFV-infected PAMs, whereas MGF110-9L deletion significantly restored host protein levels (Fig. 6F and G). Collectively, these data illustrate that pMGF110-9L plays an indispensable role in the inhibition of host protein synthesis, which is eIF2α phosphorylation- and ISR-activation-dependent.

### ASFV pMGF110-9L triggers SG formation in an eIF2α phosphorylation-dependent manner

**Fig 7:**
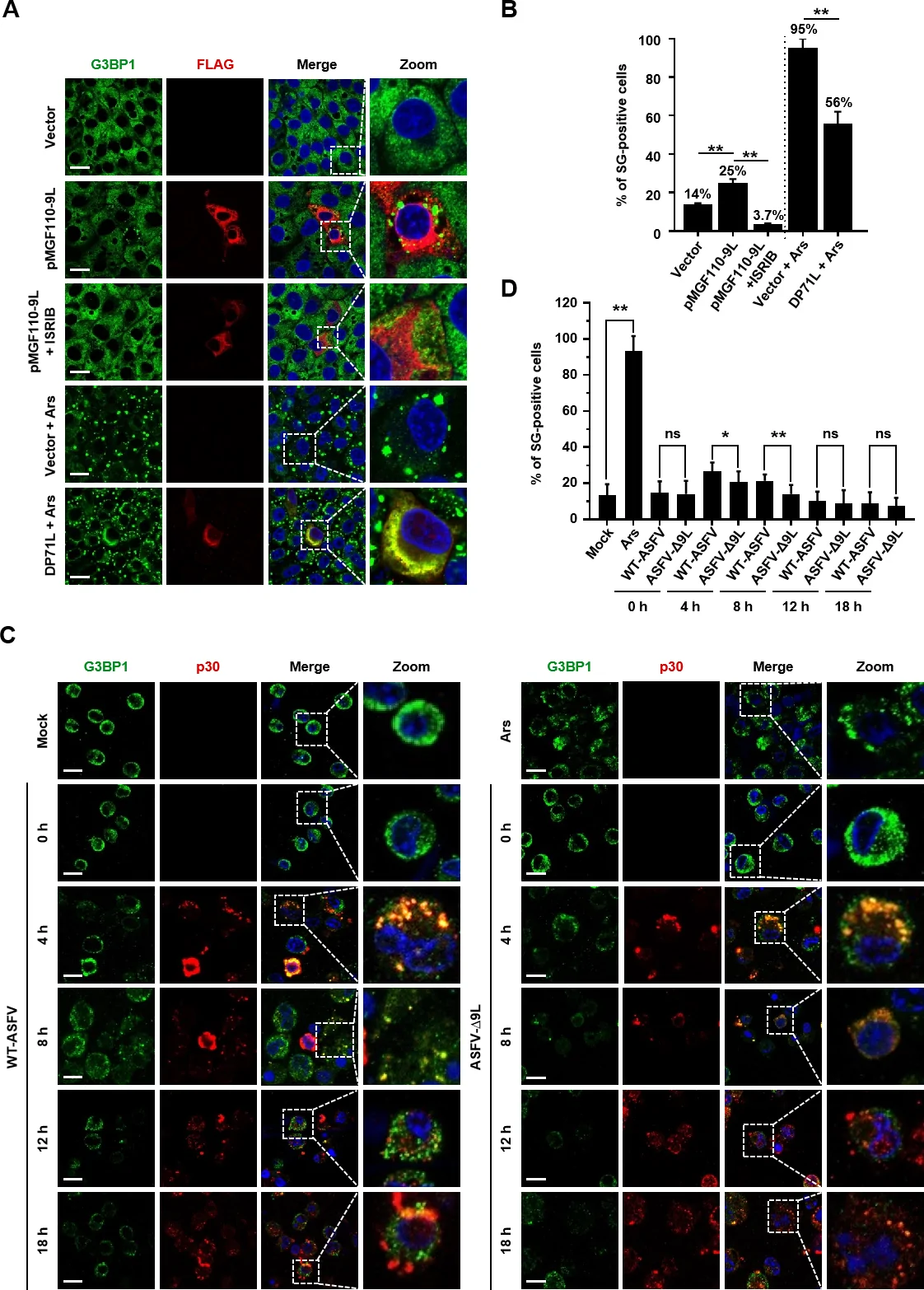
ASFV pMGF110-9L triggers SG formation. (**A**) PK-15 cells transfected with pMGF110-9L expression vector (0.5 µg) for 24 h were incubated with or without ISRIB (0.5 µM). As a control, cells transfected with the DP71L expression vector (0.5 µg) for 24 h were treated with 0.5 mM Ars for 45 min. The cells were then fixed, permeabilized, and processed for immunofluorescence assay with anti-G3BP1 (green) and anti-FLAG (red) antibodies. Nuclei were counterstained with DAPI (blue). Cells were imaged by confocal microscopy. Scale bar, 20 µm. (**B**) Representative bar plots showing the percentage of SG-positive cells calculated from 50 random fields. Data are presented as the mean ± SD of three independent experiments. **, *P* < 0.01. (**C**) PAM cells were infected with ASFV-WT or ASFV-Δ9L (MOI = 0.1). At the indicated times, the cells were fixed, permeabilized, and processed for immunofluorescence assay with rabbit monoclonal anti-G3BP1 (green) and mouse monoclonal anti-p30 (red) antibodies. Treatment with Ars served as a positive control. Nuclei were counterstained with DAPI (blue). Scale bar, 20 µm. (**D**) The percentage of SG-positive infected cells was determined by counting ≥30 random fields per condition from three independent experiments and is presented as the mean ± SD. *, *P* < 0.05; **, *P* < 0.01; ns, not significant.

A reduction in global protein synthesis is closely associated with the development of SGs and intracellular aggregates formed by stalled pretranslational initiation complexes, serving as specialized signaling platforms to intensify stress-induced translation arrest and form a unique clustering interface (51). Therefore, to address whether pMGF110-9L-triggered translational shutdown is linked to SG formation, we examined the percentage of cells displaying G3BP1-induced SGs in PK-15 cells overexpressing pMGF110-9L via confocal immunofluorescence microscopy, as previously described (52). As shown in Fig. 7A and B, Ars robustly stimulated the formation of G3BP1-induced SGs in cells by 95%, and the overexpression of pMGF110-9L significantly increased SG formation by 25%. We further used the ISR inhibitor ISRIB to assess whether pMGF110-9L-induced SG formation is linked to eIF2α phosphorylation and ISR signaling. As a positive control, ASFV pDP71L, a known eIF2α phosphorylation antagonist (41), substantially blocked Ars-induced SG formation by 56%. ISRIB led to a significant reduction (3.7%) in G3BP1-induced SGs in pMGF110-9L-expressing cells compared to untreated cells (Fig. 7A and B). Subsequently, we monitored the dynamics of SG formation in PAMs infected with WT-ASFV or ASFV-Δ9L, or treated with Ars. As expected, Ars resulted in dramatic SG formation in the PAMs. We observed a transient wave of SG formation at 4–8 hpi in WT-ASFV-infected PAMs, which gradually declined after 8 hpi and became virtually undetectable by 12–18 hpi (Fig. 7C and D). Notably, we found that ASFV-Δ9L-infected PAMs exhibited a stronger decrease in SG formation at the early stage compared to that of ASFV-WT-infected cells (Fig. 7C and D), indicating that pMGF110-9L contributes to the transient early SG formation through its ability to induce eIF2α phosphorylation. Collectively, these data demonstrate that pMGF110-9L induces SG formation in an eIF2α phosphorylation-dependent manner, and that this effect is transient and subsequently counteracted by late viral antagonists.

### TM domain of ASFV pMGF110-9L is critical for cellular translational shutdown and SG formation

**Fig 8:**
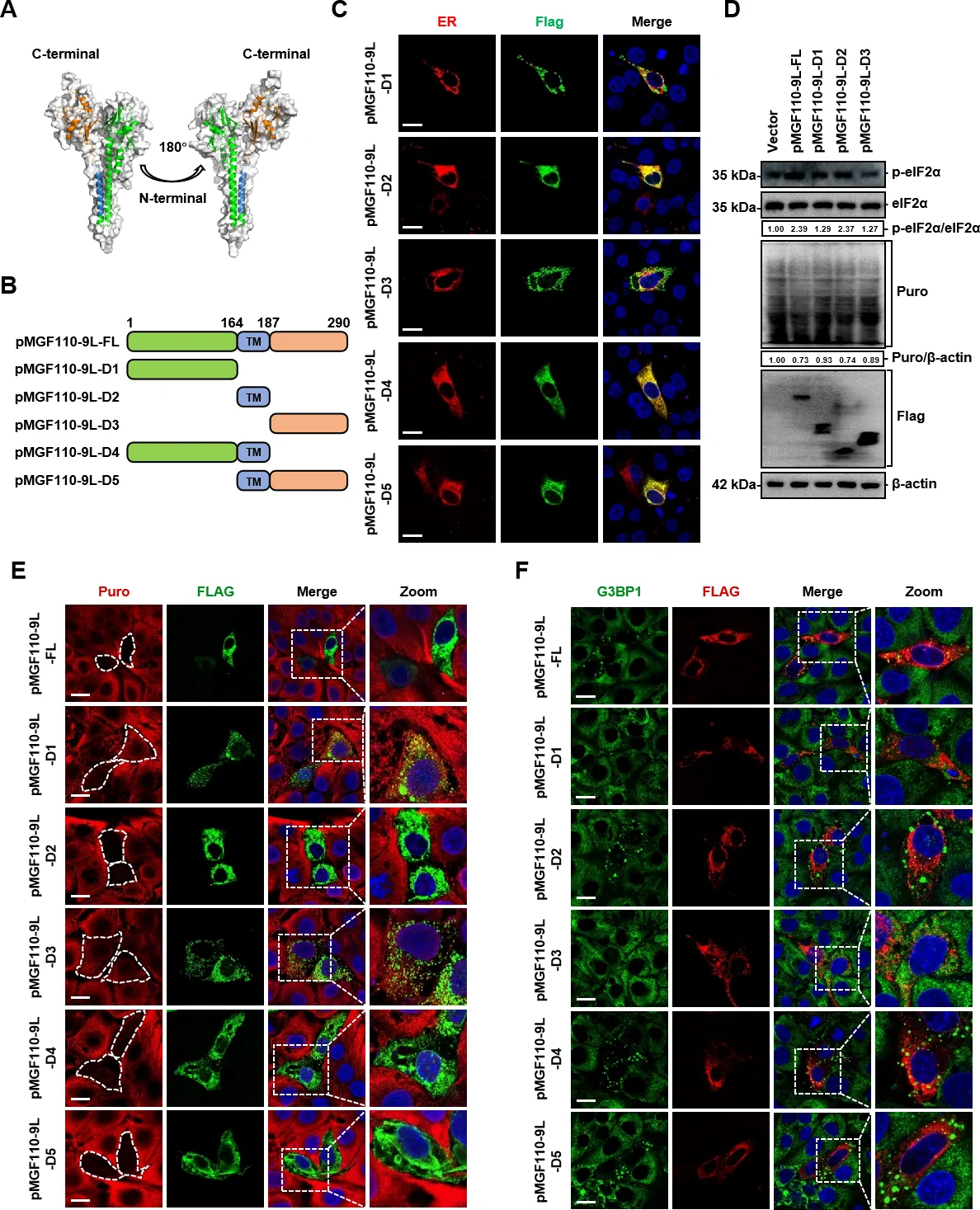
TM domain of ASFV pMGF110-9L is critical for cellular translational shutdown and SG formation. (**A**) Predicted protein structure of pMGF110-9L. Green: N-terminus. Blue: TM domain. Orange: C-terminus. (**B**) Schematic diagrams showing the truncated mutants of pMGF110-9L as follows: MGF110-9L-D1 (1–163), MGF110-9L-D2 (164–186), MGF110-9L-D3 (187–290), MGF110-9L-D4 (1–186), and MGF110-9L-D5 (164–290). (**C**) Subcellular localization of truncated pMGF110-9L in PK-15 cells. Scale bar, 20 µm. (**D**) 3D4/21 cells were transfected with an empty vector, the pMGF110-9L expression vector, or its indicated mutants (2.0 µg). At 24 h post-transfection, the cells were labeled with puromycin (5 μg/mL) for 45 min. The whole-cell lysates were then subjected to Western blot analysis. (**E**) PK-15 cells transfected with the pMGF110-9L expression vector or its indicated mutants (0.5 µg) for 24 h were labeled with puromycin for 45 min. The cells were then fixed, permeabilized, and processed for immunofluorescence assay. A FLAG-specific monoclonal antibody was used to detect pMGF110-9L or its truncated version (green). Puromycylated chains were visualized using an anti-puromycin antibody (red). Nuclei were stained with DAPI (blue). Scale bar, 20 µm. (**F**) PK-15 cells transfected with the pMGF110-9L expression vector or its indicated mutants (0.5 µg) for 24 h were processed for immunofluorescence assay with anti-G3BP1 (green) and anti-FLAG (red) antibodies. Scale bar, 20 µm.

According to the predicted protein structure from AlphaFold2 (53), pMGF110-9L contains an α-helix TM domain at its intermediate region, with a large N-terminus that projects into the organelle lumen (Fig. 8A). To elucidate the functions of these domains, five truncated mutants of pMGF110-9L were constructed: pMGF110-9L-D1 (1–163 aa), pMGF110-9L-D2 (164–186 aa), pMGF110-9L-D3 (186–290 aa), pMGF110-9L-D4 (1–186 aa), and pMGF110-9L-D5 (164–290 aa) (Fig. 8B). Truncated pMGF110-9L fragments were initially characterized by confocal immunofluorescence microscopy. The results revealed that pMGF110-9L did not colocalize with resident ER proteins following deletion of the TM domain (Fig. 8C), suggesting its critical role in anchoring the protein to the ER. Rluc assays showed that the inhibitory effects of pMGF110-9L-D1 and -D3 on Rluc expression were significantly lower than those of full-length pMGF110-9L (pMGF110-9L-FL), but that of pMGF110-9L-D2, -D4, and -D5, containing the TM domain, inhibited host gene expression, similar to pMGF110-9L-FL (Fig. S13A). Consistently, depletion of the TM domain also led to a significant decrease in the induction of eIF2α phosphorylation and ATF4 translation (Fig. 8D; Fig. S13B). Moreover, we performed a ribopuromycylation assay to determine the effects of the TM domain on pMGF110-9L-induced suppression of host protein synthesis. The results demonstrated that deletion of the TM domain negated the ability of pMGF110-9L to block host protein synthesis (Fig. 8D and E). Consistently, depletion of the TM domain also resulted in a deficiency in pMGF110-9L-induced SG formation (Fig. 8F). Taken together, these findings indicate that the TM domain of pMGF110-9L is critical for ER localization, the induction of eIF2α phosphorylation, and ISR signaling, leading to a decrease in global protein synthesis and the induction of SG formation.

## DISCUSSION

The ISR represents an important cellular response to viral infection that directs translational reprogramming and triggers a diverse set of cellular processes, such as SG assembly, autophagy, immunomodulation, metabolism, secretion, and cell cycle regulation, which are closely correlated with the induction of a protective immune response, viral replication, and virulence (16). To ensure successful replication, ASFV may deploy strategies to hijack the host translation machinery to favor viral protein synthesis and counteract innate cellular defenses (6, 43). Previous data reported that eIF2α remained unphosphorylated in productive infection of ASFV in Vero and PAM cells, while sustained eIF2α phosphorylation blocked infection in murine RAW cells, suggesting that viral modulation of the ISR partially dictates the ultimate infection outcome (43, 44, 54). Therefore, a better understanding of the mechanisms underlying the interplay between ASFV replication and host ISR will provide essential insights into viral pathogenesis and facilitate the development of MLVs.

**Fig 9:**
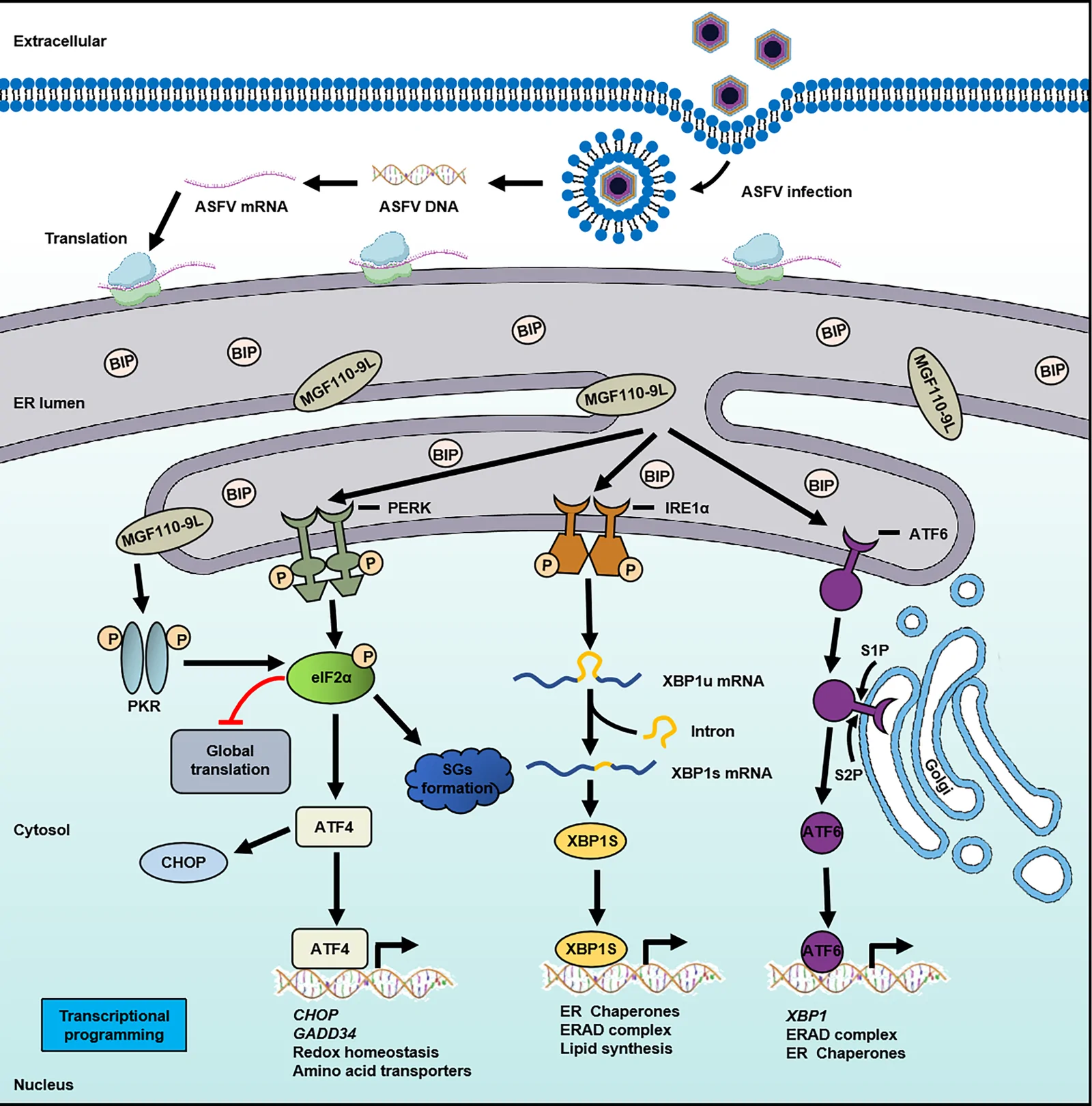
Proposed model of the involvement of pMGF110-9L in host ISR and ASFV pathogenesis. ASFV-encoded pMGF110-9L, an early ER-localized membrane protein, functions in ER swelling and aggregation, inducing ER stress and triggering the activation of the PERK-eIF2α-ATF4, IRE1α-XBP1, and ATF6 branches of the UPR. Mechanistically, pMGF110-9L activates the ISR through the PERK/PKR-eIF2α pathway, leading to a decrease in global protein synthesis and the induction of SG formation.

Herein, we systematically addressed the effects of every ASFV-encoded protein on ISR activation and identified pMGF110-9L as the strongest activator of ISR signaling, as assessed by ATF4 expression. We verified that pMGF110-9L is an ER-localized membrane protein that functions in the redistribution of resident ER proteins as well as aberrant trafficking of resident Golgi proteins from the ER, concomitant with robust ER swelling and abnormal ER morphology. Further proteomic analyses and biochemical experiments revealed that pMGF110-9L is crucial for ASFV-induced ER stress and promotes the activation of the PERK-eIF2α-ATF4, IRE1α-XBP1, and ATF6 branches of the UPR. Mechanistically, pMGF110-9L activates the ISR through the PERK/PKR-eIF2α pathway, leading to a decrease in global protein synthesis and the induction of SG formation. The TM domain of pMGF110-9L is indispensable for ER localization and ISR activation as well as for subsequent translational shutdown and SG formation (Fig. 9). Although multiple ASFV early proteins, including pMGF110-7L, pE66L, and pEP152R, have been shown to induce eIF2α phosphorylation and ISR activation (33–35), deletion of MGF110-9L alone significantly impaired ISR/UPR activation along with the accompanying translational shutdown and SG formation in the context of viral infection, indicating that pMGF110-9L plays a contributory and non-redundant role in this process. The present study provides a comprehensive understanding of how ASFV manipulates the host ISR and defines a previously uncharacterized role for pMGF110-9L in ASFV infection and pathogenesis.

After entering monocytes and macrophages, ASFV particles are transported via microtubules to the perinuclear areas within endosomes. Herein, the viral core particles enter the cytoplasm and viral DNA replication is initiated. In general, early transcripts encode proteins required for the later phase of replication, as well as those involved in immune evasion, and these early transcripts include the vast majority of MGFs. In contrast, late transcripts encode proteins required for viral morphogenesis and assembly, as well as those involved in host cell modulation (6, 7). Our genome-wide ATF4-Rluc screen shows that strong ISR activators, including pMGF110-9L and pMGF110-7L, are early-expressed ASFV proteins, whereas potent ISR inhibitors such as pDP71L are late-expressed. Consistent with this temporal pattern, Gao et al. (44) demonstrated that p-eIF2α is readily detectable at 0–2 hpi in ASFV-infected PAMs but becomes virtually undetectable from 6 hpi onward, remaining suppressed through at least 24 hpi. This early, transient wave of eIF2α phosphorylation coincides with the expression window of early viral ISR activators, such as pMGF110-9L, pMGF110-7L, and pE66L, which have been shown to activate the PERK/PKR-eIF2α pathway (34, 35). The occurrence of ER stress and UPR activation during this early window is further substantiated by recent proteomic evidence. Using 4D label-free quantitative proteomics, Zhang et al. (32) demonstrated that ASFV infection robustly activates all three branches of the UPR both *in vivo* and *in vitro*. Following this early activation phase, the subsequent disappearance of p-eIF2α signals the onset of a dominant dephosphorylation program. The late viral protein pDP71L recruits host PP1 to dephosphorylate eIF2α (37, 41, 55). Notably, *DP71L* deletion alone does not abolish eIF2α dephosphorylation (37, 41), suggesting functional redundancy among viral dephosphorylation mechanisms. Taken together, these data suggest that ASFV employs a biphasic translational manipulation strategy: early eIF2α phosphorylation triggered by viral ISR activators facilitates host translational shutoff and viral takeover, while late eIF2α dephosphorylation mediated by viral phosphatases restores global translation for mass production of late viral structural proteins. This temporally coordinated strategy may represent a hallmark of ASFV’s translational hijacking program, enabling the virus to establish favorable intracellular environments that facilitate viral propagation.

Multifunctional signaling in the ER is a major common denominator of ISR activation and is closely related to viral replication and pathogenicity (17–19). ASFV infection robustly induces ER stress and subsequently activates the PERK-eIF2α-ATF4, IRE1α-XBP1, and ATF6 branches of the UPR, which are co-opted by ASFV to facilitate viral replication (31, 32). Several ASFV proteins have been identified as being correlated with UPR signaling. Among these, pD117L activates three UPR pathways by targeting key UPR components (32). The inner membrane protein pB117L exhibits viroporin-like activity and plays crucial roles in downstream activation of the ATF6 branch and disruption of Ca²^+^ homeostasis (31). pEP152R triggers ER stress and swelling and activates the PERK/eIF2α pathway (33). The present study demonstrated that pMGF110-9L is a critical modulator of ASFV-induced ER stress and that MGF110-9L deletion strongly impaired the activation of all three UPR pathways in the context of viral infection. Notably, although ASFV-Δ9L exhibits reduced replication at late time points, the impaired ISR/UPR signaling persisted after normalization to viral p72 levels (data not shown). Together with the independent overexpression data, these findings suggest that the reduced ISR/UPR signaling in ASFV-Δ9L-infected macrophages is a direct consequence of MGF110-9L deletion rather than an indirect effect of reduced viral replication. Furthermore, pMGF110-9L-induced ER stress may contribute to PKR activation in a dsRNA-independent manner (56). Mechanistically, pMGF110-9L is an ER-localized membrane protein that not only robustly remodels ER morphology but also significantly alters protein processing, potentially disturbing ER homeostasis. This subversion appears to be linked to ER accumulation and aggresome formation, thereby disrupting proper compartment function and favoring VF formation for viral morphogenesis. These findings position pMGF110-9L as a key contributor to the host ER stress and ISR pathways that cooperate with other early viral proteins to create a replication-permissive environment, complementing the biphasic translational manipulation strategy employed by ASFV.

Additionally, ASFV manipulates other key host response pathways to promote immune evasion and pathogenesis, including interference with secretory cargo and modulation of antigen processing and presentation (6, 57, 58). Virulent ASFV infection of porcine monocytes, the primary target cells, causes a marked decrease in maturation and reduced surface transport of SLA I, which is accompanied by compromised T-cell-mediated immune defense and ASF pathogenesis. This cellular subversion is most likely due to the destruction of functional ER structures and induction of ER stress in infected cells, supplemented by a cascade of events, including global protein translation arrest, mitochondrial dysfunction, and caspase-3-mediated apoptosis (59). Our studies demonstrated that pMGF110-9L, a vital ER effector, disturbs ER homeostasis and promotes ER-associated aggresome formation. Such ER-localized aggresomes can reorganize the export compartments and secretory pathways, which may impede SLA I surface trafficking. pMGF110-9L-induced ER remodeling is also linked to ER stress and UPR, triggering the shutdown of global protein synthesis, thereby inhibiting SLA I expression. Moreover, proteomic analysis revealed the involvement of pMGF110-9L in the induction of widespread cellular stress, including ER stress, mitochondrial oxidative stress, and the shutdown of protein synthesis. Collectively, these data suggest that pMGF110-9L plays an important role in SLA I subversion of ASFV-infected monocytes.

Notably, multiple ASFV MGF110 genes, including MGF110-9L and MGF110-7L, are the top-ranked viral genes throughout infection, and several members have been implicated in ER stress induction and UPR activation (40, 60, 61). The MGF110 gene family resides within the left variable region of the ASFV genome and is subject to frequent gene duplication, deletion, and rearrangement, as commonly observed in naturally attenuated or cell culture-adapted strains, such as NH/P68, OURT 88/3, ASFV-P121, and BA71V (62–64). Large-scale deletions encompassing the MGF110 locus—including MGF110-9L—are present in macrophage-tropic and virulent isolates (e.g., Lisbon 60, Estonia 2014, and genotype I NH/P68-like strains), and MGF110 is not essential for ASFV replication in PAMs or for high-virulence phenotypes (65–67). These functions are attributed principally to the MGF360/MGF505 families (9, 68). Nevertheless, specific MGF110 members modulate early host-cell interactions. In particular, pMGF110-9L and pMGF110-7L can induce eIF2α phosphorylation, trigger ER stress and UPR branch activation, and contribute to early host translational shutoff and transient SG formation (34). Deletion of MGF110-9L in genotype II Georgia 2007-like backgrounds results in reduced early ISR activation and partial attenuation in swine (13), whereas its natural absence in other genotypes does not preclude virulence, indicating a strain-context-dependent contribution. Thus, while not a core determinant of macrophage tropism or baseline virulence, select MGF110 proteins such as pMGF110-9L and pMGF110-7L fine-tune the ISR and early viral fitness. The precise strain-specific roles of individual MGF110 members in immune evasion and pathogenesis warrant further investigation.

In summary, our study provides a comprehensive understanding of how ASFV-encoded proteins regulate the ISR. pMGF110-9L was demonstrated to be a prominent virulence factor during ASFV infection and is involved in the viral takeover of protein synthesis machinery, accompanied by ER stress, ER-associated aggresome formation, halted host protein translation, and SG formation. The TM domain of pMGF110-9L is indispensable for ER targeting. Overall, our study offers novel insights into ASFV replication mechanisms and immunoevasion strategies, highlighting pMGF110-9L as an attractive target for the development of an MLV.

## MATERIALS AND METHODS

### Cells and viruses

HEK293T (ATCC CRL-11268), PK-15 (ATCC CRL-33), and 3D4/21 (ATCC CRL-2843) cells were cultured in Dulbecco’s modified Eagle’s medium (DMEM, Solarbio, China) supplemented with 10% fetal bovine serum (FBS, Gibco, USA), 100 U/mL penicillin, and 100 μg/mL streptomycin (Gibco). PAMs isolated from specific-pathogen-free piglets were grown in RPMI 1640 medium (Solarbio) containing 10% FBS, 100 U/mL penicillin, and 100 μg/mL streptomycin. Cells were maintained at 37°C with 5% CO2. The ASFV-WT strain and ASFV-Δ9L mutant used in the present study were preserved at the LVRI and CAAS, and the ASFV-Δ9L contains only the intended deletion of MGF110-9L without additional modifications as previously described (13).

### Antibodies and reagents

Rabbit anti-eIF2α, anti-ATF4, anti-CHOP, anti-ATF6, anti-FLAG polyclonal antibodies, and mouse anti-FLAG monoclonal antibody were purchased from Proteintech (IL, USA). Rabbit anti-p-eIF2α was purchased from Abways Technology (Shanghai, China). Rabbit anti-G3BP1, anti-BIP, anti-PERK, anti-PKR, anti-IRE1α, and anti-GCN2 polyclonal antibodies; rabbit anti-p-PKR and anti-p-IRE1α monoclonal antibodies; and mouse anti-β-actin monoclonal antibody were purchased from ABclonal (Wuhan, China). Rabbit anti-p-PERK polyclonal antibody was purchased from Bioworld Technology (Nanjing, China). Rabbit anti-p-GCN2 monoclonal antibody was purchased from Abcam (MA, USA). Mouse anti-puromycin monoclonal antibody was purchased from Millipore (MA, USA). Rabbit anti-p72 polyclonal antibody and mouse anti-p30 monoclonal antibody were prepared and stored at the LVRI. The secondary antibodies conjugated to horseradish peroxidase (HRP), Alexa Fluor 488 (AF488), or Alexa Fluor 594 (AF594) were purchased from Invitrogen (CA, USA). TG, ISRIB, and GSK2606414 were purchased from MedChemExpress (NJ, USA). Sodium arsenite, puromycin, and PKR inhibitor C16 were obtained from Sigma-Aldrich (MO, USA).

### Plasmid constructs

Expression plasmids for ASFV ORFs were constructed by insertion of synthetic cDNA for a particular ORF into the pCMV vector with a C-terminal FLAG tag (Beyotime, Shanghai, China). Truncated MGF110-9L constructs were similarly cloned into the pCMV-FLAG vector. For generating the pMGF110-9L-EGFP expression vector, MGF110-9L coding sequences were cloned into the pEGFP-C1 vector (Beyotime). The ATF4-RLuc and ATF4-EGFP reporters were prepared as previously described (34). The ER marker vector pDsRed2-ER was purchased from Clontech (CA, USA).

### Luciferase reporter assays

HEK293T cells in 48-well plates were transfected with an expression plasmid for ASFV ORF (0.1 µg) and ATF4-RLuc reporter plasmid (0.05 µg), or were cotransfected with pMGF110-9L expression plasmid (0.1 µg) and pRL-TK reporter plasmid (0.1 µg). At 24 h post-transfection, the cell extracts were harvested using a passive buffer, and a luciferase reporter assay system (Promega, WI, USA) was used to determine the RLuc activities according to the manufacturer’s instructions.

### Endoplasmic reticulum isolation

PK-15 cells were transfected with the pMGF110-9L expression vector or an empty vector. At 24 h post-transfection, cells were harvested, and ER fractions were isolated using an Endoplasmic Reticulum Isolation Kit (Solarbio). The purity of the fractions was determined by immunoblotting for calreticulin (ER marker).

### Transmission electron microscopy

HEK293T and PK-15 cells were transfected with a pMGF110-9L expression vector or an empty vector. At 24 h post-transfection, cells were detached using trypsin-EDTA and collected by low-speed centrifugation. The cell pellets were fixed with 2.5% glutaraldehyde (Solarbio) for 30 min at room temperature, followed by post-fixation with 1% osmium tetroxide, dehydration through a graded ethanol series, and embedding in epoxy resin according to the manufacturer’s instructions. As a positive control, cells were treated with TG (1 µM) for 6 h prior to harvest. Ultrathin serial sections were prepared, mounted on copper mesh grids, and stained with uranyl acetate and lead citrate. Images were acquired using a transmission electron microscope (Hitachi, Tokyo, Japan).

### Western blot analysis

The transfected or virus-infected cells were washed twice with cold phosphate-buffered saline (PBS), lysed in 2× sodium dodecyl sulfate (SDS) loading buffer, and heated at 95°C for 10 min. Equal amounts of samples were separated with 8%–12% sodium dodecyl sulfate polyacrylamide gel electrophoresis (SDS-PAGE) and then transferred to polyvinyl difluoride (PVDF) membranes (Millipore). Transferred membranes were blocked with 5% fat-free milk for 1 h at room temperature. The membranes were then incubated overnight at 4°C with the primary antibodies and for 1 h at room temperature with the corresponding HRP-conjugated secondary antibodies. Finally, the membranes were visualized by a BioSpectrum 500 imaging system (UVP LLC, CA, USA). The protein bands were quantified by densitometry using the ImageJ software.

### RNA extraction and RT-qPCR

Total RNA was extracted from 3D4/21 cells using TRIzol reagent (TaKaRa, Dalian, China), according to the manufacturer’s protocols. cDNA was synthesized using a High-Capacity cDNA Reverse Transcription Kit (Vazyme, Nanjing, China). qPCR analysis was performed using ChamQ Universal SYBR qPCR Master Mix (Vazyme) on a CFX96 Real-Time PCR Detection System (Bio-Rad, CA, USA). The relative abundance of the indicated mRNA normalized to that of GAPDH was determined based on the standard 2^−ΔΔCT^ protocol. The sequences of qPCR primer pairs are listed in Table 1.

**TABLE 1:** RT-qPCR primers used in this study

Target	Forward sequence (5′–3′)	Reverse sequence (5′–3′)
Porcine ATF4	CCCTTTACGTTCTTGCAAACTC	GCTTCCTATCTCCTTCCGAGA
Porcine CHOP	CTCAGGAGGAAGAGGAGGAAG	GCTAGCTGTGCCACTTTCCTT
Porcine XBP1(s)	GAGTCCGCAGCAGGTG	CCGTCAGAATCCATGGGG
Porcine XBP1(u)	TCCGCAGCACTCAGACTACGT	ATGCCCAAGAGGATATCAGACTC
Porcine ERdj4	CAGAGAGATTGCAGAAGCATATGA	GCTTCTTGGATCGAGTGTTTT
Porcine P58IPK	GGTGCTGAATGTGGAGTAAAT	GCATGAAACTGAGATAAAGCG
Porcine EDEM1	GCATGAAACTGAGATAAAGCG	CGGCAGTGGATGGGGTTGAG
Porcine BIP	CATCACGCCGTCATATGTGG	CGTCGAAGACCGTGTTCTCA
Porcine Calreticulin	CCCACTATTTACTTCAAGGAG	GAATTTGCCGGAACTGAGAAC
Porcine GAPDH	ACATGGCCTCCAAGGAGTAAGA	GATCGAGTTGGGGCTGTGACT

### Immunofluorescence assay and confocal microscopy

The transfected or virus-infected cells were washed twice with cold PBS at the indicated times, fixed with 4% paraformaldehyde for 20 min, and permeabilized with 0.1% Triton X-100 for 15 min. After three washes with PBS, cells were blocked in 5% bovine serum albumin (Promega) in PBS for 1 h at room temperature. Cells were then incubated with primary antibodies (1:100) at 37°C, followed by Alexa Fluor secondary antibodies (1:500). Nuclei were stained with DAPI (Beyotime) for 8 min at room temperature. Cells were imaged using a Zeiss LSM800 laser-scanning microscope (Carl Zeiss, NY, USA) with a 63× oil immersion objective.

### Treatment of transfected cells with cycloheximide

HEK-293T cells were first transfected with the pMGF110-9L expression vector or empty vector for 24 h. Cells were then treated with CHX at a final concentration of 100 µg/mL for 1 h prior to transfection with the pRL-TK reporter plasmid. At 12 and 24 h post-pRL-TK transfection, cells were harvested and subjected to qRT-PCR analysis.

### Ribopuromycylation assay

The ribopuromycylation assay was performed as previously described (48). Briefly, the transfected or ASFV-infected cells were pulse-labeled with 5 μg/mL puromycin and then incubated for 45 min. As a positive control, cells transfected with an empty vector were treated with 0.5 mM arsenite for 45 min before the addition of puromycin. After three washes with PBS, the cells were prepared and subjected to immunofluorescence assay and Western blot analysis.

### Statistical analysis

All statistical analyses were performed using GraphPad Prism version 8.0.0 (GraphPad Software, CA, USA). Data were presented as the mean ± standard deviation (SD) of three independent experiments. Statistical significance was determined using an unpaired two-tailed *t*-test and assessed based on the *P*-value: *P* < 0.01 (**), *P* < 0.05 (*), ns, not significant.

## Data Availability

The authors confirm that the data supporting the findings of this study are available within the article and its supplemental material. Additional data can be obtained from the corresponding author upon reasonable request.

## SUPPLEMENTAL MATERIAL

Supplemental material jvi.01080-26-s0001.doc Fig. S1 to S13.

ASM does not own the copyrights to Supplemental Material that may be linked to, or accessed through, an article. The authors have granted ASM a non-exclusive, world-wide license to publish the Supplemental Material files. Please contact the corresponding author directly for reuse.
