# Supplementary material for: African swine fever virus pMGF110-9L plays a critical role in the integrated stress response and viral pathogenesis
Source: J Virol. 2026 Sep 8;100(10):e01080-26. doi: 10.1128/jvi.01080-26 (PMC13712873; doi:10.1128/jvi.01080-26)
Supplement: Supplementary file 1 [file jvi.01080-26-s0001.doc]

Supplementary Materials for

**African swine fever virus pMGF110-9L plays a critical role in the integrated stress response and viral pathogenesis**

Shuai Fan^1#^, Han Zhong^1#^, Dan Li^1,2#^, Jing Zhang^2^, Yuhang Zhang^1^, Man Hu^1^, Wenrui He^1,3,4^, Bo Wan^1,3,4^, Zixiang Zhu^1,2^, Shichong Han^1,3,4*^, Haixue Zheng^1,2*^, Gaiping Zhang^1,3,4*^

*Corresponding author

E-mail: hanshichong081@126.com (SCH); haixuezheng@163.com (HXZ); zhanggaiping2003@163.com (GPZ)

^#^These authors contributed equally to this work.

This PDF file includes Fig. S1, Fig. S2, Fig. S3, Fig. S4, Fig. S5, Fig. S6, Fig. S7, Fig. S8, Fig. S9, Fig. S10, Fig. S11, Fig. S12 and Fig. S13 according to the order.

**Fig. S1 Effects of ASFV-encoded proteins on the cell viability of HEK293T.**

HEK293T cells seeded in 96-well plates were transfected with an empty vector or the indicated plasmids encoding ASFV proteins (0.05 µg), respectively. At 24 h posttransfection, cell viability was measured using a CCK-8 assay. Data are presented as the mean ± SD of three independent experiments.

**Fig. S2 Expression of ASFV proteins that induced >2.5-fold upregulation of ATF4-Rluc in HEK293T cells.**

HEK293T cells were transfected with plasmids encoding the indicated ASFV proteins fused with a FLAG tag. At 24 h posttransfection, cells were fixed, stained with a mouse anti-FLAG monoclonal antibody, and visualized by indirect immunofluorescence microscopy. Scale bars, 100 μm.

**Fig. S3 Expression of ASFV proteins that induced >2.5-fold downregulation of ATF4-Rluc expression in HEK293T cells.**

HEK293T cells were transfected with plasmids encoding the indicated FLAG-tagged ASFV proteins. At 24 h posttransfection, cells were fixed, stained with a mouse anti-FLAG monoclonal antibody, and visualized by indirect immunofluorescence microscopy. Scale bars, 100 μm.

**Fig. S4. Ectopic expression of ASFV pMGF110-9L had a negligible effect on cell viability.**

HEK293T and PK-15 cells seeded in 96-well plates were transfected with pMGF110-9L expression vector with an increasing doses (0.05 µg, 0.1 µg, 0.2 µg) or an empty vector (0.2 µg). At 24 h posttransfection, cell viability was assessed with CCK-8 assays. Data are presented as the mean ± SD of three independent experiments. ns, not significant.

**Fig. S5 Effects of ASFV-WT and ASFV-Δ9L infection on the phosphorylation of eIF2α and expression of ATF4 in PAMs.**

PAMs were infected with ASFV-WT or ASFV-Δ9L at an MOI of 0.1. At the indicated times, the whole-cell lysates were collected and subjected to Western blot analysis with antibodies against p-eIF2α, total eIF2α, ATF4, p72, and β-actin (left). The band intensities of p-eIF2α (normalized to total eIF2α) and ATF4 were quantified using ImageJ software (right). Data are presented as the mean ± SD of three independent experiments. *, *P*<0.05; **, *P*<0.01; ns, not significant.

**Fig. S6 Prediction of a signal peptide in the N-terminal region of ASFV pMGF110-9L using SignalP 5.0.**

The amino acid sequence of ASFV pMGF110-9L was analyzed by SignalP 5.0 (https://services.healthtech.dtu.dk/services/SignalP-5.0/). A putative signal peptide was identified within the N-terminal 19 residues (positions 1-19), as indicated by the high S-score and the predicted cleavage site.

**Fig. S7 Multiple sequence alignment and domain analysis of ASFV pMGF110-9L from representative ASFV isolates.**

Amino acid sequences of pMGF110-9L from the indicated ASFV isolates were aligned using ClustalX 2.1, and the alignment was displayed using ESPript 3.0. Residues that are absolutely conserved among all sequences are highlighted in white on a red background. The predicted secondary structure elements and the conserved cysteine-rich region are indicated above the alignment.

**Fig. S8 Confocal immunofluorescence analysis of the intracellular distribution of ASFV pMGF110-9L protein.**

HEK293T and PK-15 cells were cotransfected with pMGF110-9L expression vector (0.5 µg) and one of the following organelle markers: pDsRed2-Mito, pDsRed2-LAMP1, or pDsRed2-Peroxi (0.2 μg). At 24 h posttransfection, the cells were fixed, permeabilized and incubated with anti-FLAG antibody and then with secondary antibody conjugated with AF488 (green). Nuclei were counterstained with DAPI (blue). The organelle marker was directly visualized (red), and localization was determined using confocal microscopy. The overlapping coefficient (R) was shown in enlarged images, and the intensity profile of the linear region of interest (ROI) across the cells contained with pMGF110-9L and the indicated organelle markers. Scale bar, 20 μm.

**Fig. S****9 Proteomics analysis of ASFV pMGF110-9L-host interactome.**

(A) Western blot and silver staining were used to detect the expression of exogenous pMGF110-9L in PK-15 cells and the enrichment of pMGF110-9L-interacting proteins. The data were tested two times independently. (B) GO enrichment analysis of all the cellular proteins interacting with pMGF110-9L. Based on the annotations from GO, these cellular proteins were categorized into different biological processes, cellular components, and molecular functions. (C) Network interactions of the pMGF110-9L-interacting proteins by STRING analysis at confidence level 0.4. The interactions between these cellular proteins are indicated by the connecting line. The thickness of the connecting line represents the strength of the associations. Proteins functionally categorized under some enriched groups based on existing information from Swiss-Prot/TrEMBL database were highlighted. (D) KEGG pathway enrichment analysis. The enriched pathways targeted by pMGF110-9L-interacting proteins were analyzed using the KEGG functional annotation pathway database.

**Fig. S10 Interaction of ASFV pMGF110-9L with ER stress-related proteins.**

(A to C) PK-15 cells were cotransfected with a FLAG-tagged pMGF110-9L expression plasmid (5 µg) and a plasmid encoding HA-tagged BIP (A), Calreticulin (B), or PDI (C) (5 µg). At 36 h posttransfection, cells were lysed and subjected to reciprocal coimmunoprecipitation (Co-IP) using anti-FLAG and anti-HA antibodies, followed by immunoblotting with the indicated antibodies to confirm bidirectional protein-protein interactions. (D to F) PK-15 cells were cotransfected with an empty vector or the FLAG-tagged pMGF110-9L expression plasmid (0.5 µg) together with a plasmid encoding HA-tagged BIP (D), Calreticulin (E), or PDI (F) (0.5 µg). At 24 h posttransfection, cells were fixed, permeabilized, and immunostained with anti-FLAG (green) and anti-HA (red) antibodies. Nuclei were counterstained with DAPI (blue). Colocalization of pMGF110-9L with the indicated ER stress-related proteins was examined by confocal microscopy. Scale bars, 20 µm.

**Fig. S11 Effects of ASFV-WT and ASFV-Δ9L infection on the activation of PERK, IRE1α, and ATF6 branches of the UPR in PAMs.**

PAMs were infected with ASFV-WT or ASFV-Δ9L at an MOI of 0.1. At the indicated times, the whole-cell lysates were collected and subjected to Western blot analysis with antibodies against BIP, p-IRE1, total IRE1, p-PERK, total PERK, and activated ATF6, p72, p30, and β-actin (left). The band intensities of BIP, p-IRE1 (normalized to total IRE1), p-PERK (normalized to total PERK), and activated ATF6 were quantified using ImageJ software (right). Data are presented as the mean ± SD of three independent experiments. *, *P*<0.05; **, *P*<0.01; ns, not significant.

**Fig. S12 Effects of ASFV-WT and ASFV-Δ9L infection on the phosphorylation of PKR in PAMs.**

PAMs were infected with ASFV-WT or ASFV-Δ9L at an MOI of 0.1. At the indicated times, the whole-cell lysates were collected and subjected to Western blot analysis with antibodies against p-PKR, total PKR, p72, p30, and β-actin (left). The band intensities of p-PKR (normalized to total PKR) were quantified using ImageJ software (right). Data are presented as the mean ± SD of three independent experiments. *, *P*<0.05; **, *P*<0.01; ns, not significant.

**Fig. S13 Effects of ASFV pMGF110-9L and its truncated mutants on TK-Rluc and ATF4-Rluc reporter gene expression.**

(A) HEK293T cells were cotransfected with pRL-TK (0.1 µg) and pMGF110-9L expression vector or its indicated mutants (0.1 µg), respectively. TG was used as the positive control. At 24 h posttransfection, the cells were harvested and subjected to the Rluc assay. (B) HEK293T cells were cotransfected with ATF4-RLuc (0.05 µg) and pMGF110-9L expression vector or its indicated mutants (0.1 µg), respectively. TG was used as the positive control. At 24 h posttransfection, the cells were harvested and subjected to the Rluc assay. Data are presented as mean ± SD of three independent experiments. *, *P*<0.05; **, *P*<0.01; ns, not significant.
